# Microbial communities related to the sulfur cycle in the Sansha Yongle Blue Hole

**DOI:** 10.1128/spectrum.01149-23

**Published:** 2023-08-25

**Authors:** Kai Sun, Min Yu, Xiao-Yu Zhu, Chun-Xu Xue, Yunhui Zhang, Xing Chen, Peng Yao, Lin Chen, Liang Fu, Zuosheng Yang, Xiao-Hua Zhang

**Affiliations:** 1 Frontiers Science Center for Deep Ocean Multispheres and Earth System, College of Marine Life Sciences, Ocean University of China, Qingdao, China; 2 Laboratory for Marine Ecology and Environmental Science, Laoshan Laboratory, Qingdao, China; 3 Institute of Evolution and Marine Biodiversity, Ocean University of China, Qingdao, China; 4 Key Laboratory of Marine Chemistry Theory and Technology, Ministry of Education, Ocean University of China, Qingdao, China; 5 Sansha Track Ocean Coral Reef Conservation Research Institute, Sansha, China; 6 College of Marine Geosciences, Ocean University of China, Qingdao, China; Institut Ruder Boskovic, Zagreb, Croatia

**Keywords:** blue hole, anoxic, sulfur cycle, microorganisms, metagenome

## Abstract

The Sansha Yongle Blue Hole (SYBH), the deepest blue hole in the world, is an excellent habitat for revealing biogeochemical cycles in the anaerobic environment. However, how sulfur cycling is mediated by microorganisms in the SYBH hasn’t been fully understood. In this study, the water layers of the SYBH were divided into oxic zone, hypoxic zone, anoxic zone I and II, and microbial-mediated sulfur cycling in the SYBH was comprehensively interpreted. The 16S rRNA genes/transcripts analyses showed that the microbial community structures associated with the sulfur cycling in each zone had distinctive features. Sulfur-oxidizing bacteria were mostly constituted by *Gammaproteobacteria*, *Alphaproteobacteria*, *Campylobacterota,* and *Chlorobia* above the anoxic zone I and sulfate-reducing bacteria were dominated by *Desulfobacterota* in anoxic zones. Metagenomic analyses showed that the sulfide-oxidation-related gene *sqr* and genes encoding the Sox system were mainly distributed in the anoxic zone I, while genes related to dissimilatory sulfate reduction and sulfur intermediate metabolite reduction were mainly distributed in the anoxic zone II, indicating different sulfur metabolic processes between these two zones. Moreover, sulfur-metabolism-related genes were identified in 81 metagenome-assembled genomes (MAGs), indicating a high diversity of microbial communities involved in sulfur cycling. Among them, three MAGs from the candidate phyla JdFR-76 and AABM5-125-24 with genes related to dissimilatory sulfate reduction exhibited distinctive metabolic features. Our results showed unique and novel microbial populations in the SYBH sulfur cycle correlated to the sharp redox gradients, revealing complex biogeochemical processes in this extreme environment.

**IMPORTANCE:**

Oxygen-deficient regions in the global ocean are expanding rapidly and affect the growth, reproduction and ecological processes of marine organisms. The anaerobic water body of about 150 m in the Sansha Yongle Blue Hole (SYBH) provided a suitable environment to study the specific microbial metabolism in anaerobic seawater. Here, we found that the vertical distributions of the total and active communities of sulfur-oxidizing bacteria (SOB) and sulfate-reducing bacteria (SRB) were different in each water layer of the SYBH according to the dissolved oxygen content. Genes related to sulfur metabolism also showed distinct stratification characteristics. Furthermore, we have obtained diverse metagenome-assembled genomes, some of which exhibit special sulfur metabolic characteristics, especially candidate phyla JdFR-76 and AABM5-125-24 were identified as potential novel SRB. The results of this study will promote further understanding of the sulfur cycle in extreme environments, as well as the environmental adaptability of microorganisms in blue holes.

## INTRODUCTION

Sulfur plays an important role in the biochemistry of marine ecosystems, which can be further linked to other important element cycles. Affected by factors such as global warming and the eutrophication of offshore waters, the dissolved oxygen (DO) content in seawater has continued to decrease recently, resulting in the rapid expansion of marine anoxic environments around the world, such as the oxygen minimum zones (OMZs) (1 – 3). Recent works on the anoxic and sulfur-rich waters of the Black Sea indicated that active microbial sulfur metabolism occurred in OMZs, and 11 phyla of sulfur-associated microorganisms were identified (4). However, due to the influence of environmental factors and human activities, the anoxic zone in waters such as the Black Sea is unstable, resulting in uncertainties in the mechanism of its sulfur cycling processes (5). As a special marine environment, oceanic blue holes have a relatively stable anaerobic water column and have been reported to harbor large numbers of microbes participating in sulfur cycling (6).

The oceanic blue hole is a special geological unit below sea level along the coastlines of islands and peninsulas, with a dark blue upper water body (7). The formation of marine blue holes first occurred during the glacial period, when the sea water level was low and carbonate rocks dissolved to form nearshore caves. During the interglacial period, when sea levels rose, limestone caves were submerged, forming marine holes filled with water (8). The currently known blue holes around the world include the Sansha Yongle Blue Hole (SYBH, 301 m) in the South China Sea, the Dean Blue Hole (202 m) in the Bahamas, the Belize Great Blue Hole (124 m) in the Caribbean Sea, and the Dahab Blue Hole (131 m) in Egypt (9, 10). The relatively enclosed geometry of blue holes results in limited exchange with other seawater, so the internal environment is usually stable (11). These blue holes have special biogeochemical features, such as the highly stratified water column (12). The temperature, salinity, DO, and other chemical factors of the water body in the cave all have chemoclines, and the bottom water is anoxic and rich in hydrogen sulfide (H_2_S) (13, 14). In anoxic environments, aerobic microorganisms are gradually replaced by facultative and strict anaerobes, normally involved in active sulfide oxidation and sulfate reduction processes (4). Based on these characteristics, blue holes can serve as natural laboratories for studying the sulfur cycle of extreme anoxic environments in marine ecosystems.

The microbial community composition and their roles in the sulfur cycle in blue holes have been investigated in recent years. For example, a study of the Jewfish Sink in the Gulf of Mexico based on the analyses of 16S rRNA gene amplicons found that there are microbial communities that potentially mediate sulfur, nitrogen, and methane metabolism in the deep anoxic layer, such as sulfur oxidizers (*Thiomicrospira* and *Sulfurimonas*) accounting for at most 37% of the total bacteria in winter (15). Studies from the Bahamas blue holes have shown that *Chlorobia*, *Arcobacter,* and *Desulfobacterota* were core members of this environment, all of which were involved in the sulfur cycle (12, 16). The metagenomic analyses of Amberjack Hole found that genes involved in thiosulfate oxidation (Sox system) were enriched in the hypoxic region, while the genes encoding enzymes of dissimilatory sulfate reduction (*sat*, *aprA,* and *dsrAB*) were enriched in anoxic layers (17, 18). These studies have provided valuable experience for exploring the physiological processes and mechanisms of microbial communities in blue holes.

The SYBH in the South China Sea is the deepest blue hole in the world and has no water exchange with the adjacent ocean (19). The physical and chemical factors of the SYBH, especially DO, showed obvious stratification and high heterogeneity (20, 21). Corresponding to the unique geochemical characteristics, the microbial community structure of the SYBH has an apparent vertical stratification, which is closely related to the DO and H_2_S gradients of the SYBH (22, 23). Previous studies have explored the abundance and diversity of microbial communities in the SYBH based on 16S rRNA gene amplicons, and found that sulfide-oxidizing bacteria (SOB) in the SYBH included green sulfur bacteria (GSB), purple sulfur bacteria (PSB), purple non-sulfur bacteria (PNSB), and colorless sulfur bacteria (CSB), among which CSB and PNSB were dominant between 100 and 140 m, while sulfate-reducing bacteria (SRB) were dominant in deeper water layers (6, 24). However, the sulfur-cycling-related metabolic potential of microorganisms therein has not yet been well elucidated.

In this study, we collected seawater samples from 29 different depths (0 m to 190 m) of the SYBH for 16S rRNA genes/transcripts sequencing and seven depths for metagenome sequencing. The composition and distribution of microbial communities involved in the sulfur cycle in the SYBH were examined based on 16S rRNA amplicons to elucidate the interaction of microbial communities and environmental factors in depth. In addition, 16S rRNA transcripts were analyzed for the first time, demonstrating that sulfur-associated microorganisms were not only highly abundant in the SYBH but also active. Moreover, the vertical distribution patterns of sulfur metabolic genes and the main groups of SOB and SRB were determined by metagenomic analyses. This investigation reveals the diversity of sulfur-associated microorganisms in the SYBH and potential mechanisms driving the sulfur cycling in marine anoxic environments.

## RESULTS

### Physical and chemical profiles of the SYBH

**Fig 1:**
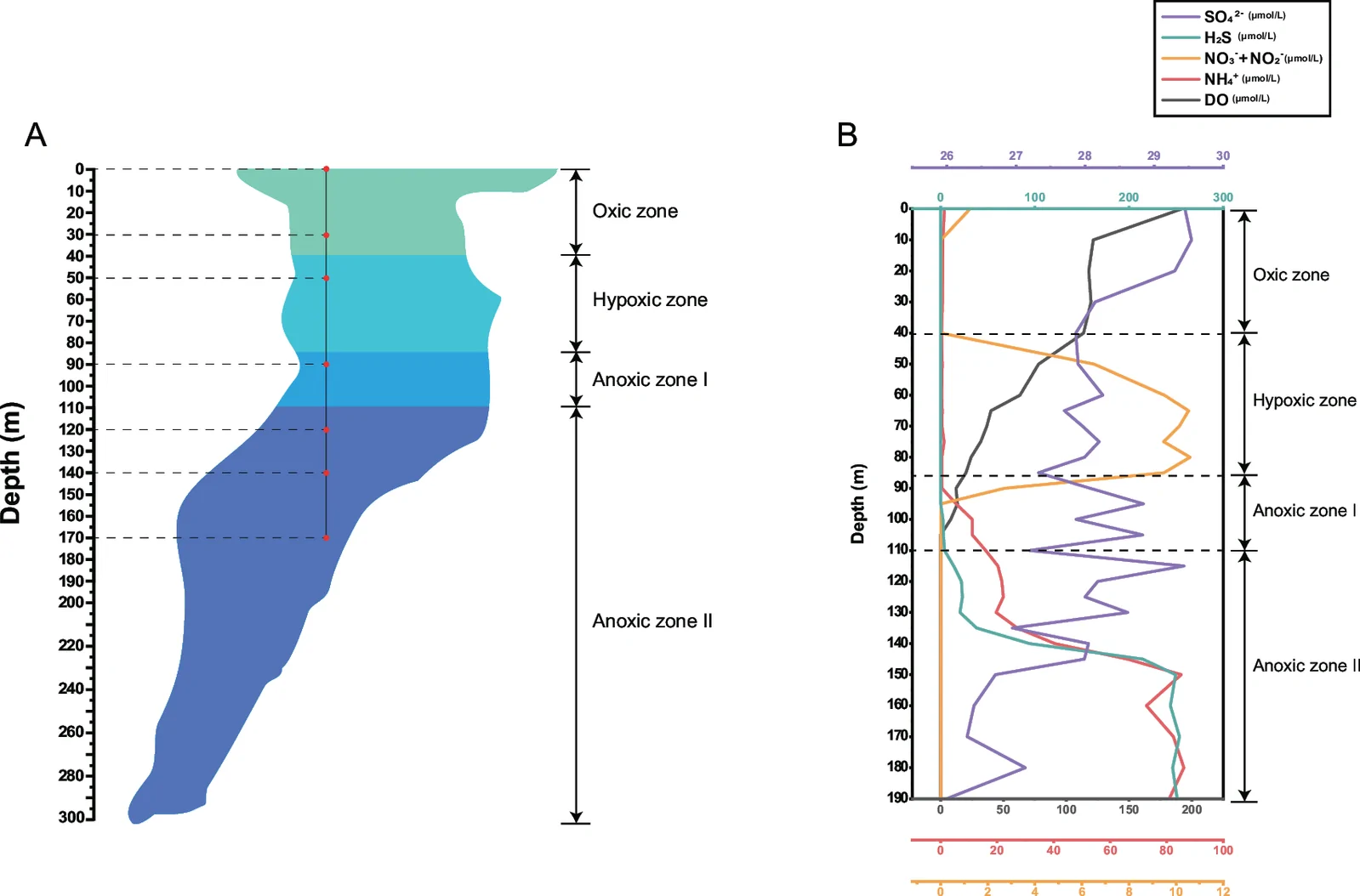
The morphological map and environmental profile of the SYBH. (**A**) The vertical geometry of the SYBH (red dots indicate the metagenomic sampling depths). The water layers are divided into oxic zone, hypoxic zone, anoxic zone I, and anoxic zone II based on the oxygen concentrations. (**B**) Concentrations of different environmental parameters along the depths at the SYBH.

DO in the SYBH reduced from 192 µmol/L at the surface (0 m) to 8 µmol/L at 100 m and dropped to nearly 0 µmol/L at 105 m. The water of the SYBH was highly stratified according to DO and was divided into oxic zone (0–40 m), hypoxic zone (50–85 m), anoxic zone I (90–110 m), and anoxic zone II (115–190 m) (Fig. 1A and B; Table S1). In addition, the environmental parameters of the seawater samples, such as dissolved inorganic nitrogen (NO_3_^−^ and NO_2_^−^), ammonium (NH_4_^+^), sulfate (SO_4_^2-^), and H_2_S also showed significant stratifications at different depths (Fig. 1B). The concentration of SO_4_^2−^ was at a high level in the oxic zone (reaching the highest value of 29.54 µmol/L at 10 m) and decreased to a similar level of that in the hypoxic zone. The SO_4_^2−^ concentration fluctuated sharply in the anoxic zone I and decreased significantly in the anoxic zone II. H_2_S concertation began to increase in the anoxic zone I (90 m below), which showed a sharp elevation at 130–150 m and remained at a high level of ~250 µmol/L below 150 m in the anoxic zone II. The variation trend of NH_4_^+^ concertation was similar to that of H_2_S. NO_3_^−^ and NO_2_^−^ concertation began to increase at 40 m and showed the highest concentration in the hypoxic zone.

### Microbial community composition and sulfur metabolic groups in the SYBH

A total of 2,096,476,770 high-quality 16S rRNA genes and 175,305,600,296 16S rRNA transcripts sequences were obtained from the 115 seawater samples (with 25,111 sequences for each sample after the random rarefaction; Table S2). A total of 27,507 operational taxonomic units (OTUs) were classified based on a 97% sequence similarity. The microbial community in the SYBH was mainly composed of *Proteobacteria*, *Bacteroidota*, *Desulfobacterota*, *Planctomycetota*, *Chloroflexi*, SAR406 clade, *Actinobacteriota*, *Firmicutes*, *Patescibacteria,* and *Latescibacterota*, which accounted for 47.92%–92.26% of the total sequences in different samples (Fig. S1). *Proteobacteria* and *Bacteroidota* were the dominant phyla of the total microbial communities across all samples. Except for F100, *Bacteroidota* was the dominant group in the anoxic zone I, with the highest relative abundance of 47.66% (P95). The relative abundance of *Desulfobacterota* obviously increased in anoxic zones, with the highest abundance reaching 37.81% (F150). The 16S rRNA transcripts showed a similar distribution pattern of the microbial community across the water column compared with that based on 16S rRNA genes.

**Fig 2:**
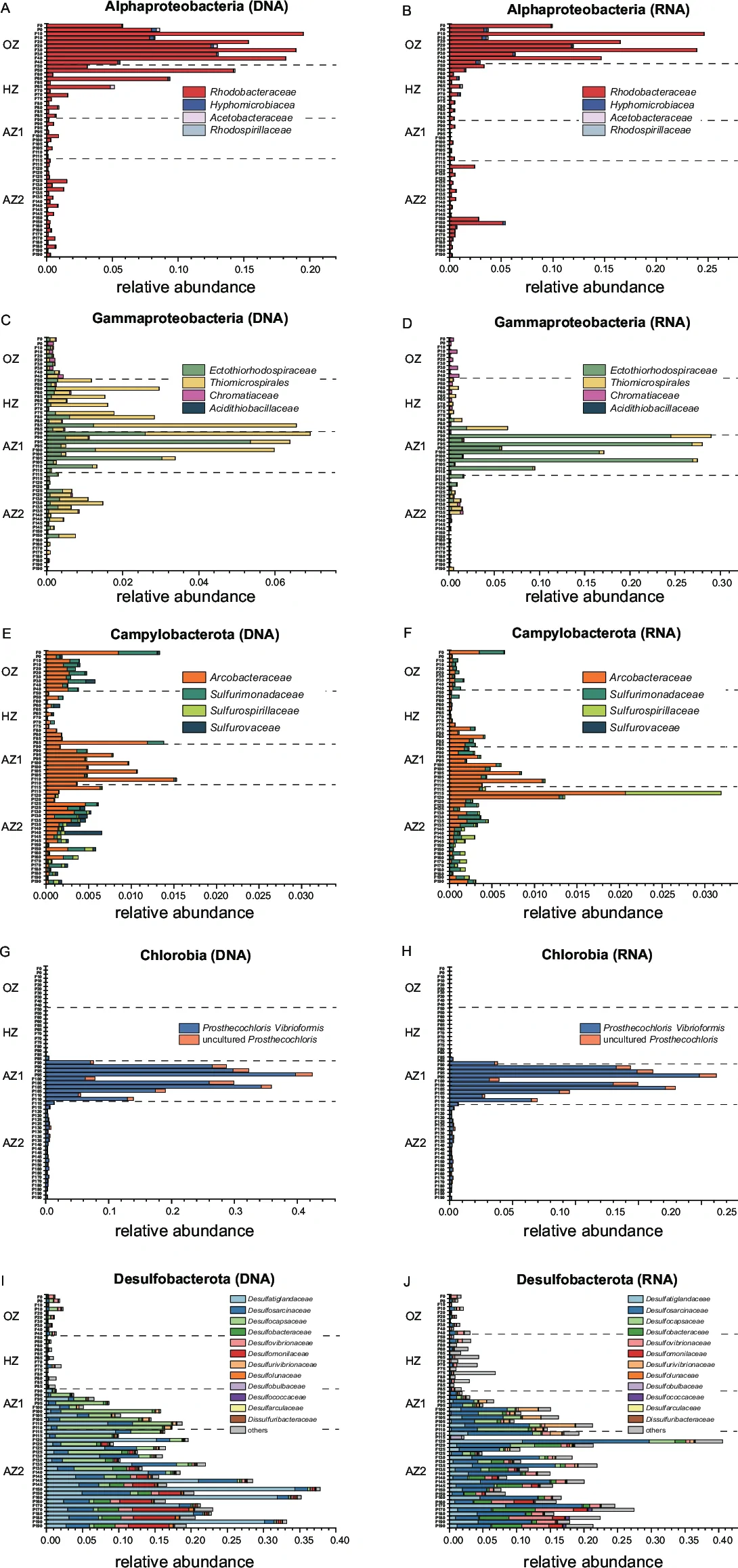
Community compositions of sulfur-associated microorganisms in the SYBH. Sample names are defined by sampling depth and lifestyle. Relative abundance of sulfur-oxidizing taxa in *Alphaproteobacteria* (**A, B**), *Gammaproteobacteria* (**C, D**), *Campylobacterota* (**E, F**), *Chlorobia* (**G, H**), and sulfate-reducing taxa in *Desulfobacterota* (**I, J**) are shown. OZ, oxic zone; HZ, hypoxic zone; AZ1, anoxic zone I; AZ2, anoxic zone II.

Besides all OTUs, a total of 1,115 SOB and SRB OTUs were selected for further analyses. The relative abundance of known SOB and SRB communities is shown at the order/family levels in Fig. 2, and the results based on DNA and RNA showed a similar trend. Major SOB species in the SYBH belonged to *Alphaproteobacteria*, *Gammaproteobacteria*, *Campylobacterota,* and *Chlorobia* (Fig. 2). *Rhodobacteraceae*, *Hyphomicrobiaceae*, *Acetobacteraceae,* and *Rhodospirillaceae* in *Alphaproteobacteria* were identified as PNSB (25) and were mainly distributed in the oxic zone (Fig. 2A and B), among which *Rhodobacteraceae* was dominant. *Ectothiorhodospiraceae* and *Chromatiaceae* of *Gammaproteobacteria* in the SYBH belonged to PSB which can oxidize thiosulfate and sulfide (26). *Ectothiorhodospiraceae* was the dominant SOB in *Gammaproteobacteria* with a rather higher relative abundance in RNA samples (at most 26.94% of the total community), whereas *Thiomicrospirales*, which belonged to CSB, was dominant both in the hypoxic zone and anoxic zone I (Fig. 2C and D) (27). The relative abundance of active *Chromatiaceae* was slightly higher in the oxic zone than that of total *Chromatiaceae*. Generally, the abundances of the above taxa were higher in free-living (FL; 0.22–3 µm) samples compared with particle-associated (PA; 3–20 µm) samples. In addition, other CSB groups like *Arcobacteraceae*, *Sulfurimonadaceae*, *Sulfurospirillaceae,* and *Sulfurovaceae* from the phylum *Campylobacterota* were mainly active in the anoxic zone I (Fig. 2E and F) (28). *Prosthecochloris*, the only GSB in the SYBH, was enriched in the anoxic zone I (Fig. 2G and H), which can oxidize H_2_S and S_0_ under anaerobic conditions (29, 30).

The major SRB species belonged to *Desulfobacterota*, whose relative abundance displayed an obviously increasing trend below 90 m, and were consistently high in anoxic zones (Fig. 2I and J). At the family level, the dominant groups were *Desulfatiglandaceae*, *Desulfocapsaeae*, *Desulfosarcinaceae*, *Desulfomonilaceae*, *Desulfobacteraceae,* and *Desulfovibrionaceae*. Interestingly, these groups showed different distribution patterns in the anoxic zone, hinting at their different ecological niches. For example, in the active communities, the relative abundance of *Desulfosarcinaceae* increased significantly in anoxic zones and that of *Desulfatiglandaceae* and *Desulfocapsaceae* decreased accordingly.

### Diversity of total and active microbial communities in the SYBH

**Fig 3:**
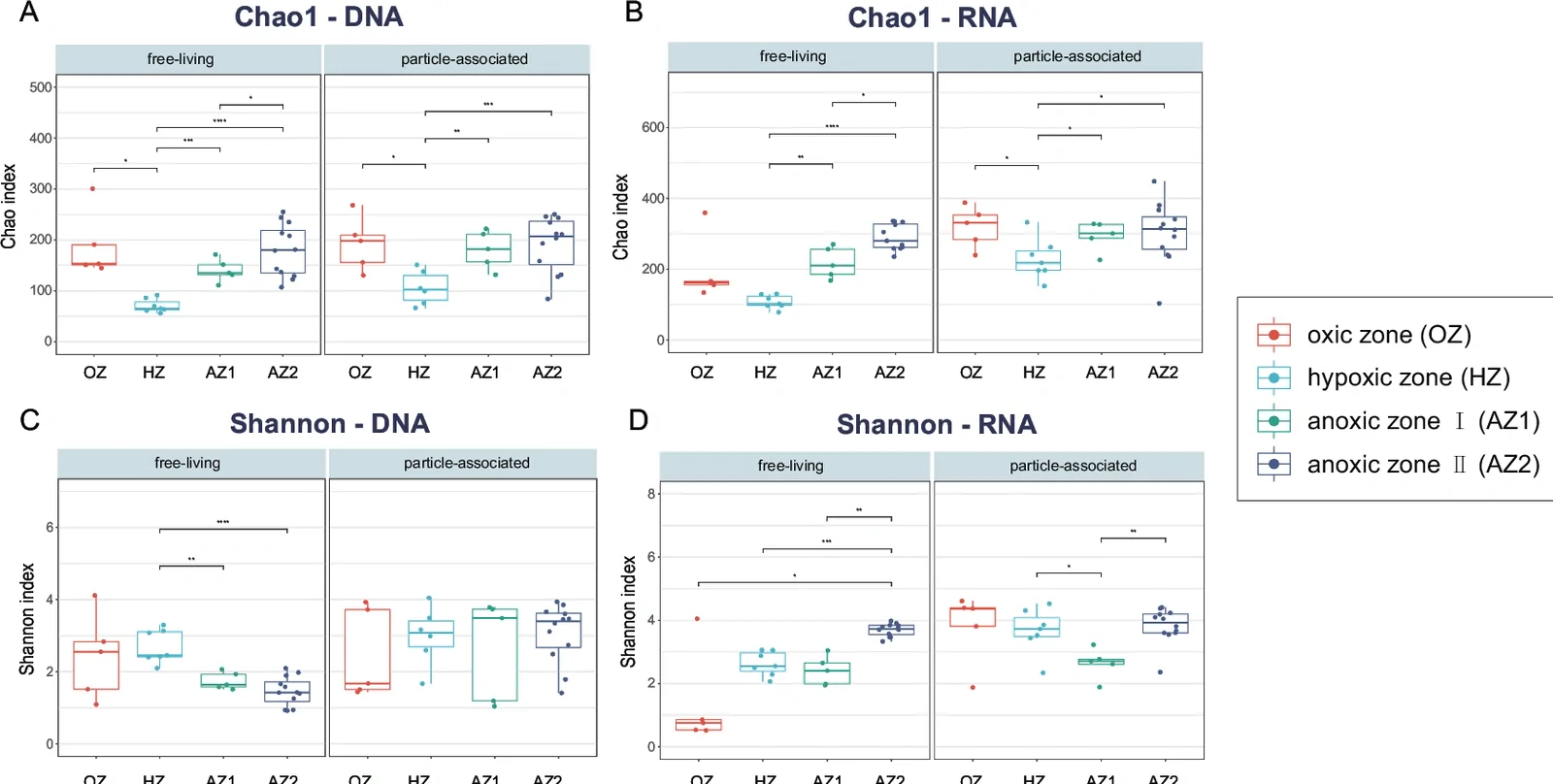
Chao1 and Shannon indices among the sulfur-associated microorganisms by depth and lifestyle in the SYBH. Chao1 on (**A**) DNA and (**B**) RNA levels; Shannon on (**C**) DNA and (**D**) RNA levels. The between-group variation adopts the *t* test (*P < 0.1; **P < 0.01; ***P < 0.001).

The alpha diversity indices Chao 1 (reflecting richness) and Shannon (reflecting diversity) were calculated. The rarefaction curves of the Shannon index of total OTUs indicated that the amount of sequencing data could reflect the diversity of the microbial community (Fig. S2). The richness and diversity of total OTUs were clearly stratified across depths, and both indices showed similar trends (Fig. S3). It is worth mentioning that the diversity of active PA microbial communities in the anoxic zone I was significantly higher than that in other water layers. As for SOB and SRB OTUs, the richness of FL and PA microbial communities possessed a similar pattern on both the DNA and RNA levels (Fig. 3A and B). The richness decreased from the oxic zone to the hypoxic zone and then generally increased with depth to the anoxic zone II, indicating abundant microbes involved in sulfur cycling in anoxic zones of the SYBH. For the diversity, analyses of DNA and RNA showed different results (Fig. 3C and D). From the perspective of active microorganisms, the diversity of SOB and SRB in the FL samples was the lowest in the oxic zone and peaked in the anoxic zone II, while that in PL samples was comparable between zones except for the anoxic zone I where the lowest value was observed.

**Fig 4:**
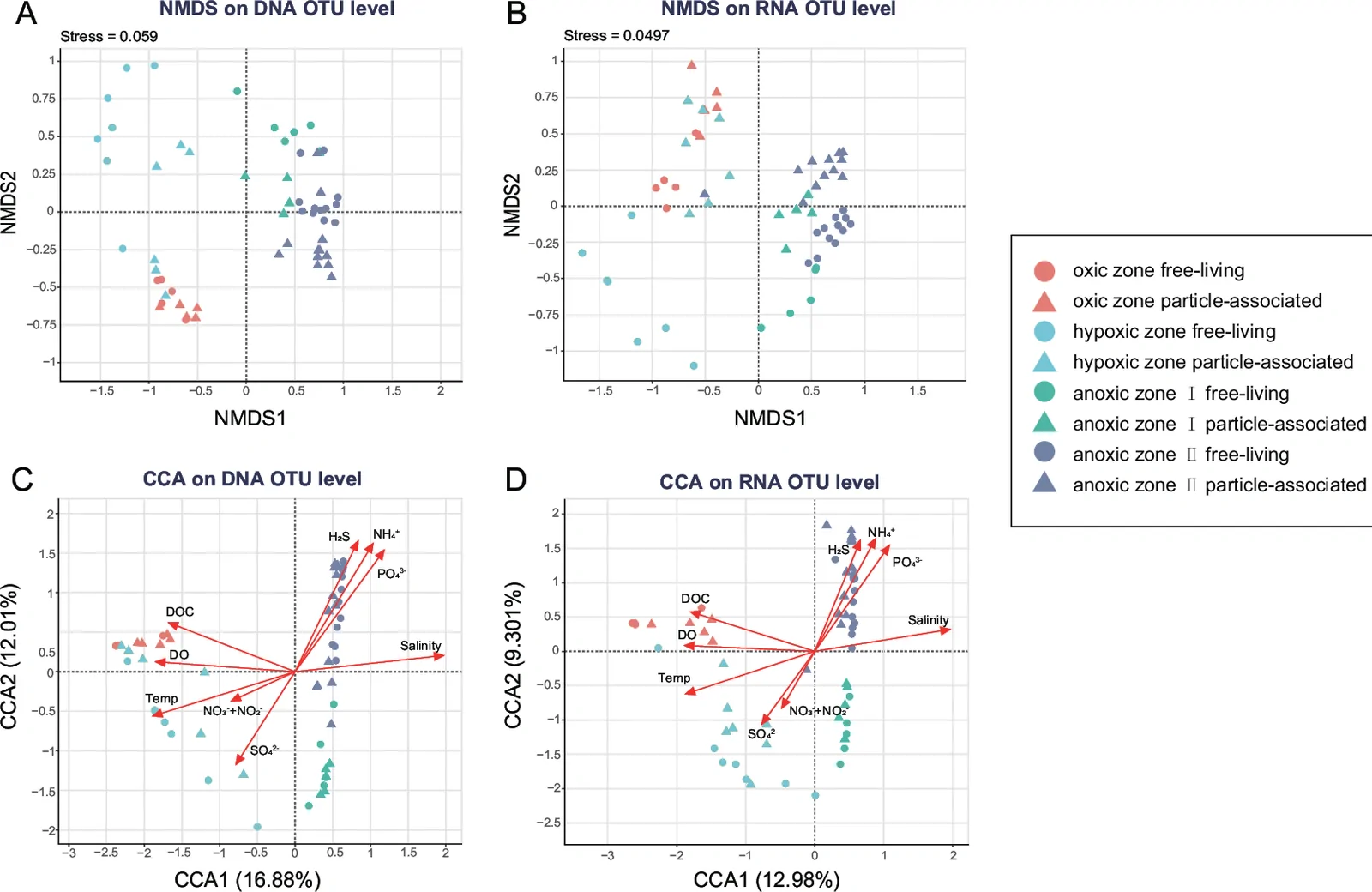
Community dissimilarity analysis among the sulfur-associated microorganisms in the SYBH at the OTU level. (**A, B**) Non-metric multidimensional scaling (NMDS) analysis illustrating the differences between groups. The distance algorithm adopts Bray-Curtis dissimilarity, and the between-group variation adopts Adonis. (**C, D**) Canonical correspondence analysis (CCA) illustrates the relationships between the microbiology community and environmental factors. Temp, temperature; DO, dissolved oxygen; DOC, dissolved organic carbon.

For both total prokaryotes and sulfur-associated bacteria, distinct communities between four zones and different lifestyles (FL and PA) were observed on the DNA and RNA levels (Fig. 4A and B; Fig. S4A and B), consistent with the results of community composition analysis (Fig. 2; Fig. S1). The total microbial communities were influenced by multiple environmental factors, and the association of SOB and SRB with environmental factors was similar to that of the total communities in the SYBH (Fig. 4C and D; Fig. S4C and D). For sulfur-associated microbes, DO [*r^2^* (DNA) = 0.8329, *r^2^* (RNA) = 0.8522] and DOC [*r^2^* (DNA) = 0.7620, *r^2^* (RNA) = 0.8370] were the main factors separating the populations of the oxic zone from those of the deeper zones. The sulfur-associated microbes of the hypoxic zone were separated from other groups by high concentrations of NO_3_^−^, NO_2_^−^ [*r^2^* (DNA) = 0.1930, *r^2^* (RNA) = 0.2148] and SO_4_^2-^ [*r^2^* (DNA) = 0.4594, *r^2^* (RNA) = 0.4025]. The sulfur-associated microbes in the anoxic zone II were mainly influenced by high levels of H_2_S [*r^2^* (DNA) = 0.7809, *r^2^* (RNA) = 0.6924], NH_4_^+^ [*r^2^* (DNA) = 0.8532, *r^2^* (RNA) = 0.7966] and PO_4_^3-^ [*r^2^* (DNA) = 0.8752, *r^2^* (RNA) = 0.8269].

### The distribution of microbial sulfur metabolic genes across the water column

**Fig 5:**
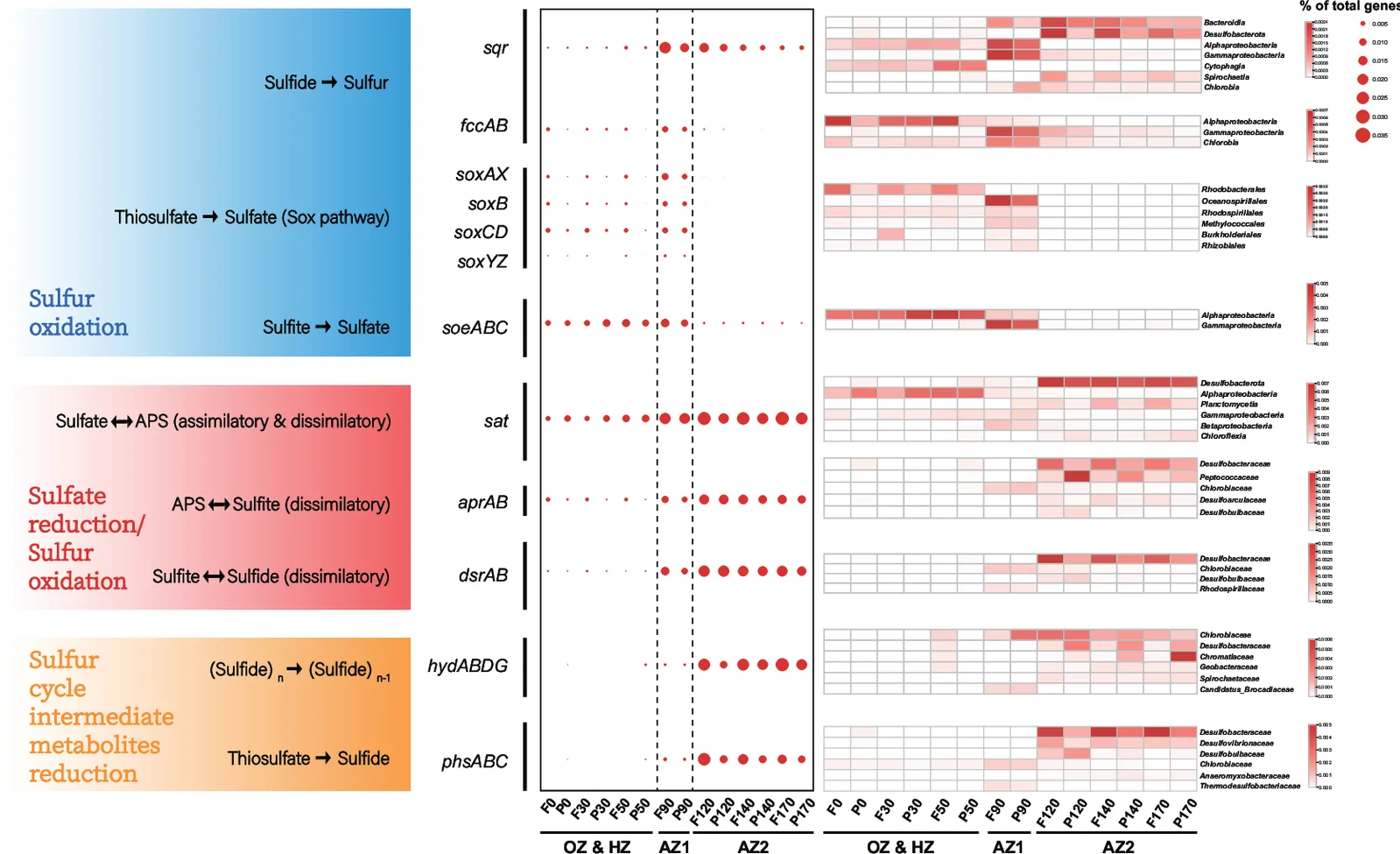
Sulfur-associated metabolic potential in the metagenomes. Bubble plot shows the relative abundances of sulfur-associated genes at different depths. Heatmap shows the community composition of these genes at different samples. OZ, oxic zone; HZ, hypoxic zone; AZ1, anoxic zone I; AZ2, anoxic zone II; *sqr*, sulfide:quinone oxidoreductase; *fccAB*, flavocytochrome c sulfide dehydrogenase; Sox family, sulfur-oxidizing multienzyme complex; *soeABC*, sulfite dehydrogenase; *sat*, sulfate adenylyltransferase; *aprAB*, adenylylsulfate reductase; *dsrAB*, dissimilatory sulfite reductase; *hydABDG*, sulfhydrogenase; *phsABC*, thiosulfate reductase.

The distributions and relative abundances of functional genes related to sulfur oxidation, sulfate reduction, and the reduction of other sulfur cycle intermediate metabolites were analyzed. The sulfur oxidation genes were generally more abundant above 90 m except *sqr* and genes for sulfate and other intermediate metabolites reduction were at high levels in layers below 90 m (Fig. 5). The abundances of genes involved in sulfate reduction were highly consistent with the abundance of SRB and the increase of H_2_S.

The sulfide:quinone oxidoreductase (Sqr) encoding gene *sqr*, which catalyzes the oxidation of sulfide to elemental sulfur, was mainly detected in anoxic zones (Fig. 5). However, genes *fccAB* encoding for the flavocytochrome c sulfide dehydrogenase (Fcc) (31), which also participates in the oxidation of sulfide to elemental sulfur, were more abundant in the oxic-hypoxic zone and anoxic zone I. Additionally, other genes related to the Sox multienzyme complex (SoxAX, SoxB, SoxCD, and SoxYZ) responsible for the complete oxidation of thiosulfate (S_2_O_3_^2−^) to SO_4_^2−^ (32, 33), as well as sulfite dehydrogenase (SoeABC) involved in the oxidation of SO_3_^2−^ (34), were mainly found in the oxic-hypoxic zone and anoxic zone I. Moreover, microbial groups containing these sulfur-oxidizing genes were distinct between the oxic-hypoxic zone and anoxic zone I (Fig. 5). For instance, genes of the SoeABC and Sox system were mainly found in *Rhodobacterales* at the oxic-hypoxic zone and in *Oceanospirillales* at the anoxic zone I.

The dissimilatory sulfate reduction (DSR) pathway is a common way for all known SRB to perform sulfate reduction (35, 36). As expected, genes including *sat* (sulfate adenylyltransferase), *aprAB* (adenylylsulfate reductase), and *dsrAB* (dissimilatory sulfite reductase) were mainly distributed in *Desulfobacteraceae* at the anoxic zone II. In addition, based on phylogenetic analysis, the reverse DsrAB (rDsrAB), which is responsible for the oxidation of S_0_ for energy (37, 38), basically existed in the anoxic zone I and belonged to *Alphaproteobacteria*, *Gammaproteobacteria*, *Betaproteobacteria,* and *Chlorobia* (Fig. S5 and S6). Genes *hydABDG* encoding for sulfhydrogenase were mainly detected in the anoxic zone II. Genes *phsABC* encoding for thiosulfate reductase, which can convert thiosulfate to sulfite and H_2_S, was most abundant in the anoxic zone II (39).

### Identification of potential SOB and SRB MAGs

**Fig 6:**
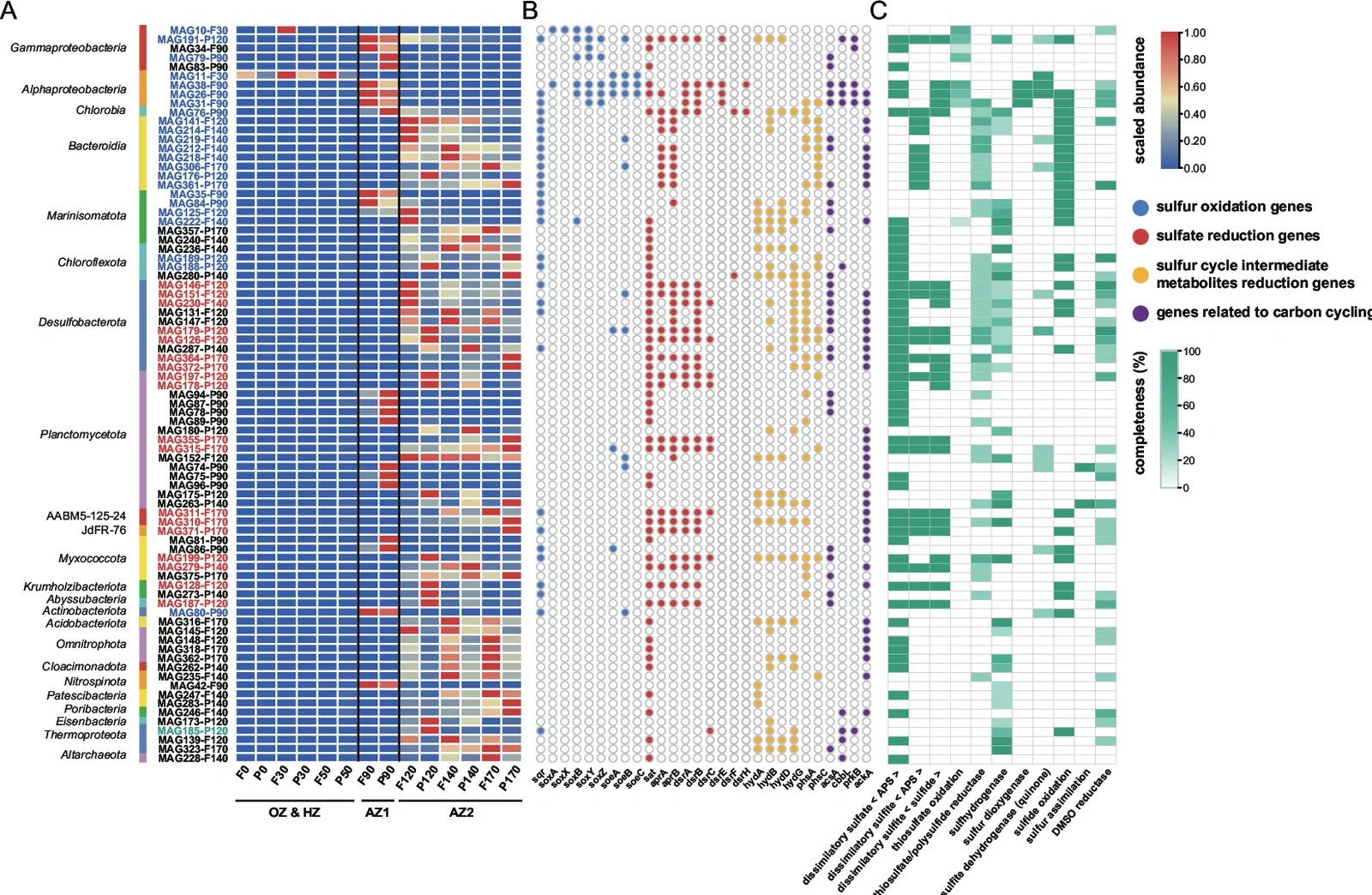
A taxonomic and genetic overview of potential SOB and SRB MAGs affiliated with 21 phyla in the SYBH. SOB MAGs are marked in blue, SRB MAGs are marked in red, and a sulfur-oxidizing archaeal MAG is marked in green. (**A**) Scaled abundance of potential SOB and SRB MAGs at different water depths. Heatmaps in (**A**) were illustrated using the Zero-to-One method at each MAG scale using TBtools, setting the maximal relative abundance of each MAG to one. OZ, oxic zone; HZ, hypoxic zone; AZ1, anoxic zone I; AZ2, anoxic zone II. (**B**) Functional sulfur-associated gene distribution of potential SOB and SRB MAGs. (**C**) Pathway completeness of sulfur cycle function in the SYBH.

From the 14 metagenomic samples in the SYBH, a total of 108 non-redundant metagenome-assembled genomes (MAGs) were recovered, representing 26 known phyla (Fig. S7). MAGs belonging to sulfur cycle-related strains were determined based on the presence of at least one gene related to sulfur metabolism (*sqr*, Sox system, *soeABC*, *sat*, *aprAB*, *dsrAB, hydABDG,* and *phsABC*). We finally screened out 81 sulfur-associated MAGs, which were affiliated with 21 phyla, including 24 sulfur-oxidizing MAGs and 18 sulfate-reducing MAGs (Fig. 6). The archaeal MAG185, which had the gene *sqr*, was classified as *Thermoproteota*. The MAGs for SOB were sorted into six taxa consisting of *Gammaproteobacteria* (3), *Alphaproteobacteria* (4), *Bacteroidota* (9), *Marinisomatota* (4), *Chloroflexota* (2), and *Actinobacteriota* (1). The SOB of *Bacteroidota* were divided into *Chlorobia* (1) from the anoxic zone I and *Bacteroidia* (8) generated from the anoxic zone II. The selected MAGs distributed in the oxic zone were only MAG10 (*Burkholderiales* in *Gammaproteobacteria*) and MAG11 (*Puniceispirillales* in *Alphaproteobacteria*). Among these MAGs, genes encoding rDsrA were identified in *Gammaproteobacteria*, *Alphaproteobacteria,* and *Chlorobia*. Besides, the abundance of MAGs belonging to *Alphaproteobacteria* in FL samples was higher than that in PA samples. Moreover, other SOB of *Bacteroidia*, *Chloroflexota,* and archaeal MAG185 (*Thermoproteota*) were mainly distributed in anoxic zone II. Dominant SRB MAGs were affiliated with *Desulfobacterota* (7), *Planctomycetota* (4), AABM5-125-24 (2), JdFR-76 (1), *Myxococcota* (2), *Krumholzibacteriota* (1), and *Abyssubacteria* (1). All SRB MAGs from this study were enriched in anoxic zone II.

Previous phylogenetic inferences suggested that the candidate phyla AABM5-125-24 (MAG310, MAG311) and JdFR-76 (MAG371) might be affiliated with the Fibrobacteres-Chlorobi-Bacteroidetes (FCB) superphylum, and the JdFR-76 was previously affiliated with KSB1 (40, 41). Both AABM5-125-24 and JdFR-76 belonged to anaerobic heterotrophic bacteria, and some genomes in AABM5-125-24 were involved in the sulfur cycle but their metabolic characteristics in the SYBH are not clear (41, 42). A total of 31 genomes of KSB1 and 26 genomes of AABM5-125-24 were collected from the National Center for Biotechnology Information (NCBI) database for the comparative genomic analysis (Fig. S8A). The reference MAGs of JdFR-76 were mainly obtained from hydrothermal vent environments, suggesting the bacteria belonging to JdFR-76 from blue holes and hydrothermal vents might share similar genomic features. The MAGs of AABM5-125-24 were divided into two clades according to different sampling environments and metabolic abilities, and MAGs in our study were clustered in clade II. The MAGs of clade I in AABM5-125-24 were isolated from groundwater and wastewater, whereas MAGs from clade II were broadly distributed, including freshwater, marine sediment, hydrothermal vents, and hypoxic seawaters (Fig. S8A).

The metabolic functions of MAGs from candidate phyla JdFR-76 and AABM5-125-24 were further investigated (Fig. S8B). The genes of complete dissimilatory sulfate reduction (*sat*, *aprAB,* and *dsrAB*) were present in MAG371, whereas genomes of JdFR-76 collected from other environments lacked these genes. Genes *acsA* and *ackA*, encoding acetyl-CoA synthetase and acetate kinase, were key genes related to acetate metabolism (43, 44). Both *acsA* and *ackA* were detected in JdFR-76 MAGs; however, almost all MAGs of JdFR-76 contain *ackA*, whereas only some MAGs contained *ascA*. Moreover, only one MAG in JdFR-76 contained nitrate reductase (*narGHI*). The complete dissimilatory sulfate reduction pathway was present in MAGs of AABM5-125-24 clade II, which was absent in the clade I. *acsA* was found in both clades of AABM5-125-24, while *ackA* existed only in the clade II. In addition, MAGs in AABM5-125-24 contained most genes associated with denitrification (*nar*, *nir*, *nor,* and *nos*) and dissimilatory nitrate reduction (DNRA, *nrf*).

## DISCUSSION

The sharp redox gradient and anoxia condition resulted in high concentrations of sulfates and sulfides in the water column of the SYBH, which indicated that SRB and SOB might be core members in this environment (45, 46). The microbial communities that dominated the SYBH have been studied previously. However, studies primarily based on 16S rRNA gene amplicon sequencing and analysis cannot reflect the activity of the microbial communities (6, 22). Meanwhile, prior to the present study, the biogeochemical sulfur cycle in the SYBH had not been fully explored (24). Importantly, the metabolic potentials and ecological roles of the enriched SRB and SOB in the SYBH were not well discussed. This study first demonstrates the distribution and activity of sulfur-associated microorganisms in the SYBH by analyzing the 16S rRNA genes and transcripts. The remarkable diversity and taxonomic novelty of sulfur-associated microorganisms in the SYBH were revealed by mining MAGs from the SYBH. Moreover, metabolic functions of the novel sulfur-associated microbial communities distinct from other marine environments were identified in the SYBH.

In this study, we demonstrated that there were strong selections of microorganisms by characterizing the diversity and taxonomic composition of the microbial communities in the water layers at depths of 0–190 m in the SYBH. As typical anoxic environments, the physicochemical properties of water bodies in the SYBH were similar to the Black Sea. DO approached 0 µmol/L at 70 m and sulfide began to accumulate at 105 m in the Black Sea (47). The abundance of SOB in the Black Sea peaked at the anoxic zone (105 m), with *Thiopontia* belonging to *Gammaproteobacteria* (4, 48) as the most abundant group while SRB gradually increased in abundance at the anoxic zone (90 m), which was consistent with that of the SYBH. Similar to the situation in the Bahamas blue hole, *Chlorobia*, *Arcobacter,* and *Desulfobacterota* were also enriched in the water column of the SYBH, indicating that the sulfur-associated microbial communities and metabolic functions are somewhat consistent across different blue holes (12, 16).

The distribution patterns of dominant phyla found in this study were basically consistent with those in previous studies (22, 49, 50). For example, there was little change in the abundance of PNSB from *Alphaproteobacteria* in the SYBH compared with the previous study (6), indicating the temporal stability of PNSB in the oxic zone. The final product of PNSB participating in sulfide oxidation is sulfate, and the tolerance of PNSB to sulfide is weaker than other SOB. Therefore, *Rhodobacteraceae*, the dominant group of PNSB in the SYBH (Fig. 2A and B), was mainly distributed in the oxic zone (51). For the two main PSB groups in *Gammaproteobacteria*, *Chromatiaceae* was mainly distributed in the oxic zone, while *Ectothiorhodospiraceae* was mainly distributed in the hypoxic zone and deeper water column, and the relative abundance was much higher than that of *Chromatiaceae* (Fig. 2C and D). The causes of this phenomenon may be different sulfide oxidation mechanisms of these two groups, and the intermediate metabolite sulfur globules of *Ectothiorhodospiraceae* are deposited extracellularly, while the protein-coated sulfur of *Chromatiaceae* is stored in the periplasmic space (52, 53). However, it is worth noting that, contrary to previous studies, the abundance of *Chlorobia* and *Desulfobacterota* increased significantly, indicating that these two taxa may play important roles in the anoxic sulfur biogeochemical cycle in the SYBH (6, 22, 24). *Desulfobacterota* can apparently be involved in most sulfate reduction, and its distribution was consistent with the concentration of H_2_S (Fig. 1B; Fig. 2I and J). It can be determined that *Desulfobacterota* as a typical SRB showed excellent adaptive capacities in the unique anoxic environment in the deep blue hole (54, 55). In this study, 16S rRNA transcripts amplicon sequencing was used for the first time to explore the distribution of microbial communities in the SYBH. Compared with gene samples, the abundance of active *Ectothiorhodospiraceae* and *Desulfosarcinaceae* increased in the anoxic zone I and anoxic zones, respectively, indicating that they play important ecological roles in specific water layers.

The richness and diversity of FL and PA microbes in anoxic marine environments have been reported in previous studies (22, 56 – 58). However, there were slight differences between the stratification of the SYBH water column in this study and the previous report based on DO (6), which may be caused by different sampling seasons. Similar to the previous study, the richness of microbial communities in the oxic zone and the anoxic zone II was higher than that in the hypoxic zone and the anoxic zone I (Fig. S3A and B) (6). DO and high concentrations of SO_4_^2−^ and H_2_S in the SYBH were important factors affecting microbial communities. Based on this, we further found that different depths harbored distinct dominant SRB and SOB, probably due to the selection and adaptation in different sulfur compound concentrations (59). Therefore, the richness and diversity of SOB and SRB communities were analyzed exclusively. From the hypoxic zone to the anoxic zone II, the microbial richness increased gradually (Fig. 3A and B), indicating that the high concentration of H_2_S provided the material basis for the growth and metabolism of sulfur-oxidizing microorganisms (60). Furthermore, according to the non-metric multidimensional scaling and canonical correspondence analysis, it was found that the sulfur-associated microbial communities at different depths clustered separately, with the communities in the oxic-hypoxic zone positively correlated with DO, SO_4_^2−^, NO_3_^−^ and NO_2_^−^ (Fig. 4). Based on this phenomenon and the high abundance of SOB, we presumed that microbially mediated sulfide oxidation and nitrate reduction reactions might be coupled in the hypoxic zone of the SYBH (61). Microbial communities in anoxic zones were positively correlated with H_2_S, NH_4_^+^ and PO_4_^3−^, indicating that there may be a syntrophic relationship between ammonia-oxidizing bacteria with SRB (62).

There was obvious stratification of sulfur-containing compounds in the SYBH, especially the enrichment of sulfide in the anoxic zone, which could be used as the key energy source for SOB in anoxic zones. The end product of the sulfur oxidation process was sulfite or sulfate, and the sulfur elements and thiosulfate acted as intermediate metabolites, which were produced by different SOB communities and functional genes (46, 63). The genes *sqr* and *fccAB*, which could catalyze the oxidation of sulfide, were distributed in different water layers (Fig. 5). The *sqr* was mainly distributed in anoxic zones and *fccAB* was distributed in the anoxic zone I and above water layers, and the abundance of *sqr* was significantly higher than that of *fccAB*. Moreover, most of the assumed SOB obtained from the SYBH contained *sqr*, while *fccAB* was not annotated in these genomes. It was reported that Sqr had a strong affinity for high concentrations of sulfide, whereas FccAB reacted with low concentrations of sulfide, which may explain why *sqr* dominated the sulfur oxidation of the SYBH in the high-sulfide environment (64). The Sox system and SoeABC were mainly dominant in *Rhodobacterales* at the oxic-hypoxic zone and *Oceanospirillales* of *Gammaproteobacteria* at the anoxic zone I (Fig. 5), revealing that these specific SOB groups may mediate radical thiosulfate and sulfite oxidation in various ways. Notably, *Oceanospirillales* is a kind of chemolithotrophy, and the MAGs of *Oceanospirillales* from the Hypersaline Great Salt Lake showed minimal genes responsible for the Sox system, which indicated that different ecological environments may affect whether *Oceanospirillales* possessed complete thiosulfate oxidation capacity (65). The sulfate pool, combined with the high concentration of organic matter in the anaerobic environment, provides a large amount of substrate for heterotrophic SRB and produces inorganic sulfide for SOB (66). Consistent with the distribution of the SRB community, genes of the DSR pathway mainly existed in *Desulfobacterota* at anoxic zones of the SYBH (Fig. 5). In summary, the vertical distribution patterns of sulfur-associated genes were investigated in the SYBH in-depth, and the results were generally consistent with previous studies in the SYBH and the Amberjack Blue Hole from the Gulf of Mexico (17, 24). It suggests that the sulfur metabolism driven by microorganisms in blue holes is the key biogeochemical process and novel microbial communities involved in the sulfur cycle in the SYBH and their functions need to be further explored.

**Fig 7:**
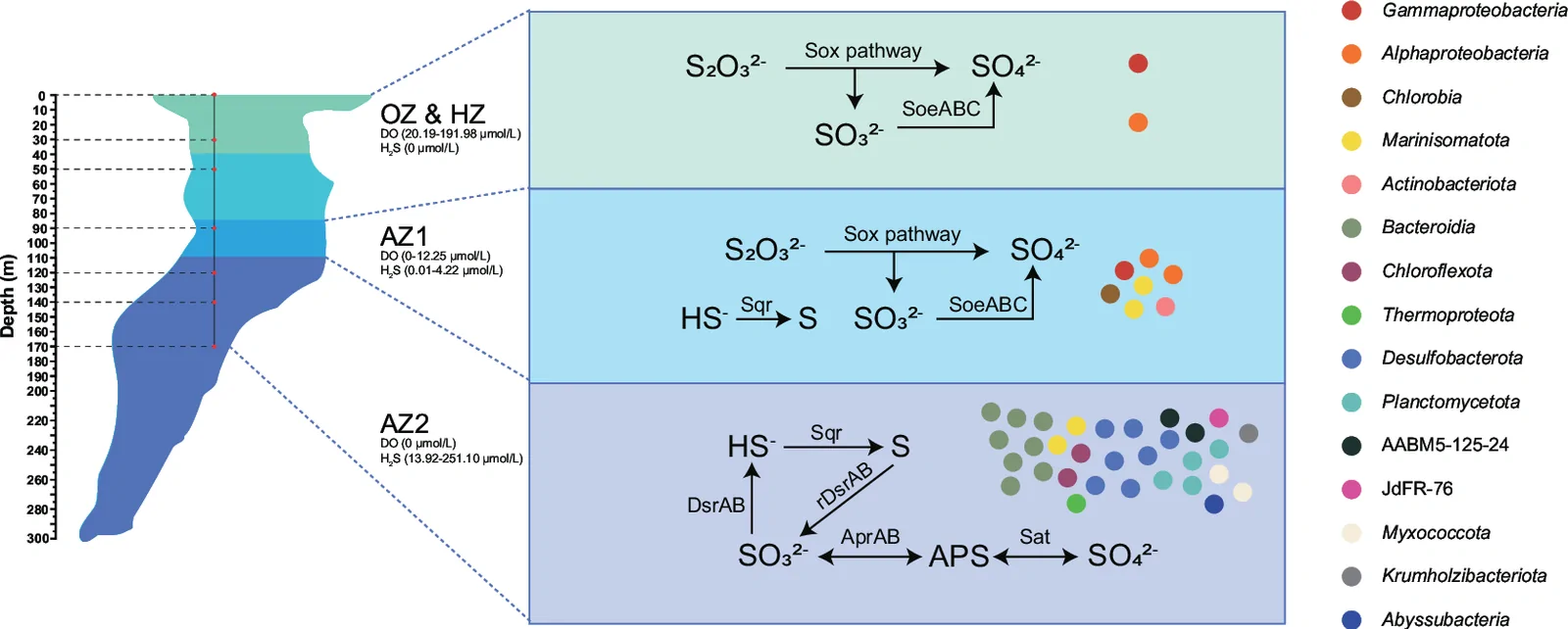
The sulfur-associated metabolic pathways of microbial MAGs in different water layers. Each point represents a genomic MAG with the color corresponding to its taxonomy. OZ, oxic zone; HZ, hypoxic zone; AZ1, anoxic zone I; AZ2, anoxic zone II.

In this study, we obtained a large number of SRB and SOB MAGs (40) in the water column of the SYBH to decipher their role in the anoxic environment (Fig. 6). In the previous metagenomic analyses of the Amberjack Hole, only 20 MAGs related to sulfur cycle were obtained (belonging to *Desulfobacterota*, *Actinobacteriota*, *Gammaproteobacteria*, *Alphaproteobacteria*, *Bacteroidota*, *Thermoplasmatota*, *Myxococcota,* and *Marinisomatota*), and no novel candidate phylum has been discovered among these MAGs (17). This indicates that the sulfur-associated taxa in the SYBH may be more abundant than that in the Amberjack Hole with an unstable anaerobic condition. The SOB MAGs were distributed in all water layers in the SYBH, and related taxa mediated thiosulfate and sulfite oxidation in the oxic-hypoxic zone and anoxic zone I, while sulfide oxidation mainly occurred in anoxic zones. SRB MAGs were distributed only in the anoxic zone II and mediated dissimilatory sulfate reduction (Fig. 7). The distribution pattern of key SOB (*Gammaproteobacteria*, *Alphaproteobacteria*, *Chlorobia,* and *Bacteroidota*) and SRB (*Desulfobacterota* and *Planctomycetota*) MAGs was similar with the results of 16S rRNA genes/transcripts analyses. As a poorly studied SOB in *Gammaproteobacteria*, except for the Sox system, MAG79 (*Pseudomonadales*) also encodes *narGHI* in the denitrification pathway, which is consistent with the MAGs assigned to *Pseudomonadales* in the sediment of Siberian soda lake, reflecting the coupling mechanism of sulfur oxidation and nitrate reduction under anaerobic environments (67). The sulfur oxidation capacity of SOB MAGs belonging to *Bacteroidia*, *Marinisomatota*, *Chloroflexota,* and *Actinobacteriota* was identified in other environments such as seafloor sulfide deposits, OMZs, groundwater, and geothermal environments (68 – 71). The archaeal MAG185 belongs to *Thermoplasmatota* and its potential sulfide-oxidizing capacity has been found in the novel *Thermoplasmatota* clade from deep-sea sediments (72). DsrEFH can act as an effective sulfur donor for DsrC to participate in the sulfur oxidation process, and genes encoding DsrEFH were annotated in several SOB MAGs (73). In addition to typical SRB *Desulfobacterota*, other groups such as *Planctomycetota*, *Myxococcota,* and *Abyssubacteria* have all been discovered in previous studies to carry genes suggestive of dissimilatory sulfate reduction (74 – 77). It is worth mentioning that *Krumholzibacteriota* has only been observed to mediate assimilation sulfite reduction through the ASR system (75). Moreover, the presence of *sqr* in some SRB MAGs suggested that they may use sulfide generated by dissimilatory sulfate reduction to obtain energy (78).

The novel SRB species were identified in the SYBH, which expands our current understanding of the blue hole as a reservoir of diverse sulfur-associated microorganisms. Genomic analyses suggested that FCB superphylum are capable of mediating key steps in biogeochemistry such as sulfur and nitrogen cycling (40, 79), and JdFR-76 and AABM5-125-24 might be affiliated with the FCB superphylum. AABM5-125-24 and JdFR-76 were mainly distributed in anoxic environments, and some genomes in AABM5-125-24 were identified to possess dissimilatory sulfate reduction and nitrite ammonification abilities (41, 42). Nevertheless, the unique metabolic mechanisms of JdFR-76 and AABM5-125-24 involved in the sulfur cycle and adapted to anaerobic environments have not been thoroughly investigated. In the reported JdFR-76 genomes, the complete dissimilated sulfate reduction pathway has only been identified in MAG371 (Fig. S8B), which may reflect that the distinctive ecological environment of the SYBH confers specific metabolic capabilities on microbial groups. Acetate was likely assimilated in the MAGs of JdFR-76 and AABM5-125-24, as evidenced by the presence of *ackA* and *acsA*, which are capable of catalyzing the bidirectional transformation of acetyl-CoA and acetate (59, 80). The correlation between *sqr* and *acsA* in MAGs of JdFR-76 suggests that acetate and hydrogen sulfide may jointly provide the energy and materials for the growth and development of JdFR-76. In the comparative genomic analysis of AABM5-125-24, the two clades were significantly different in metabolic capacities (Fig. S8B), which indicated that the versatile metabolisms of AABM5-125-24 occupied different niches. AABM5-125-24 was also regarded as a potential SRB in the Black Sea, which is the same as in the SYBH, indicating that AABM5-125-24, as a new candidate, may play an important role in the sulfate reduction in anoxic marine environments (4). Our results provide valuable experience for enriching the diversity and metabolic functions of microorganisms related to the sulfur cycle in the SYBH.

### Conclusion

In this study, we investigated the diversity, activity, and metabolic functions of microbial communities associated with sulfur cycling in the SYBH. The special physicochemical properties and geographical isolation make the microbial community composition in the SYBH unique. We identified distinct hierarchical vertical profiles of the major SOB and SRB communities in the SYBH based on 16S rRNA genes/transcripts amplicons analyses and revealed for the first time the distribution of active microbial communities. The functional genes involved in the sulfur cycle of the SYBH and their representative microbial taxa were analyzed through metagenomic analyses, and diverse SOB and SRB were detected. Finally, three MAGs belonging to the candidate phyla JdFR-76 and AABM5-125-24 were selected for further study. These MAGs showed unique metabolic functions compared with genomes isolated from other marine extreme environments, and these two groups were identified as potential novel SRB. Overall, the blue hole can be used as an important model for studying biogeochemical cycles, especially the sulfur cycle in marine anoxic environments. In the future, it will be necessary to isolate and culture SOB/SRB strains to help understand the environmental adaptation of microorganisms in the SYBH.

## MATERIALS AND METHODS

### Sampling and analysis of environmental factors

Seawater samples from 29 water layers (spanning from 0 to 190 m, with 5 or 10 m as an interval) were obtained by Niskin bottles in the SYBH (111° 768′ E, 16° 525′ N) during 24–25 October 2019. To compare the 16S rRNA gene abundance and diversity between microbial communities with FL and PA lifestyles, the seawater samples (1 L) in Niskin bottles from each sampling depth were pooled and subsequently filtered through 3 µm (TSTP, 142 mm, Millipore) and 0.22 µm (GTTP, 142 mm, Millipore) polycarbonate membranes. Microbial samples collected on 3 µm filters were designated as PA microbes, while samples collected on 0.22 µm filters were defined as FL microbes. Seawater samples from 0, 30, 50, 90, 120, 140, and 170 m were chosen for further metagenomic sequencing (Fig. 1A). Fifty liters of seawater were taken from each depth and subsequently filtered through 3 µm and 0.22 µm polycarbonate membranes. All filters for DNA or RNA extraction (1 mL RNAlater was added for preventing RNA degradation) were stored in liquid nitrogen until DNA/RNA extraction. Samples were named based on their size and depth (F0, P0, F30, P30, F50, P50, F90, P90, F120, P120, F140, P140, F170, and P170; letters “F” and “P” represent samples collected on 0.22 µm and 3 µm polycarbonate membranes, respectively). Salinity and temperature were determined with a conductivity-temperature-depth profiler (Seabird SBE 19plus, USA) before sampling. DO was measured on-site using the conventional Winkler titration method (81). Sulfide (expressed as total H_2_S) was also determined on-site using a spectrophotometer at 670 nm after color development (82). Samples for dissolved inorganic nutrients (nitrate, nitrite, ammonium, phosphate, and silicate) and sulfate were filtered with 0.45 µm cellulose acetate membranes and stored at −20°C and 4°C, respectively. Nutrients were analyzed with colorimetric methods, using an AA3 Auto-Analyzer (Seal Analytical, UK) (21). Sulfate was analyzed using ion chromatography (ICS-3000, Dionex, USA) on 1:800 diluted aliquots in Milli-Q water (83).

### DNA, RNA extractions, and sequencing

DNA and RNA for amplicon sequencing were extracted by the DNeasy PowerSoil Pro Kit (Qiagen, Germany) and RNeasy PowerSoil Total RNA Kit (Qiagen, Germany) according to the manufacturer’s instructions, respectively. The DNA and RNA samples were sequenced by Majorbio Bio-pharm Technology Co., Ltd. (Shanghai, China). The V4 regions of the 16S rRNA genes/transcripts were amplified using the primer pairs 515F and 806R (84) and then sequenced by Illumina MiSeq PE300 (MiSeq Reagent Kit v3) platform. The metagenomic DNA of each sample was extracted as previously described using the phenol-chloroform extraction method (85). Briefly, the polycarbonate membranes were washed by extraction buffer [100  mM Tris-HCl (pH 8.0), 100  mM sodium EDTA (pH 8.0), and 100  mM sodium phosphate (pH 8.0), 1.5 M NaCl, 1% CTAB] to gain biomass. The biomass was concentrated after centrifugation, ground within liquid nitrogen, and incubated within proteinase K at 37°C and SDS at 65°C, respectively. After being extracted by phenol-chloroform and precipitated by isopropanol, the DNA samples were dissolved in TE buffer and sent to BGI (BGI, Wuhan, China) for metagenomic sequencing. A 300–400 bp DNA fragment library was constructed by the BGI sequencing platform and then sequenced after the library was qualified.

### Community structure and diversity analyses using DNA- and RNA-based 16S rRNA gene amplicon sequences

The paired-end reads generated from the Illumina Miseq PE300 platform (MiSeq Reagent Kit v3) were processed by trimming the barcodes and primers and then merged using Fast Length Adjustment of Short Reads (FLASH version 1.2.11) (86). Reads shorter than 50 base pairs with an average quality score lower than 20 and with any ambiguous bases were removed. USEARCH7-uparse was used to cluster the clean data into OTUs, with a 97% similarity cutoff (87). The most common sequences in each OTU were selected as representative sequences. Taxonomic assignments were annotated with the SILVA databases (version 138) for bacteria and archaea (88). Community richness Chao1 and diversity Shannon were calculated with Mothur (version 1.30.2) (89). NMDS analysis was used to demonstrate dissimilarities between different samples by Quantitative Insights into Microbial Ecology (QIIME) (version 1.9.1) based on Bray-Curtis dissimilarity and Adonis analysis, and CCA was performed to explore the correlations between environmental factors and microbial communities with QIIME (version 1.9.1) (90).

### Metagenome assembly and functional annotation

High-quality clean data were obtained after removing the low-quality sequences and adaptors in the raw metagenomic sequencing data by fastp (91). For each sample, clean reads were then assembled into contigs larger than 500 bp using MEGAHIT (Table S3) (92). Encoding genes of prokaryotes in metagenomic samples were predicted by MetaGeneMark (93). High-quality gene catalogs were constructed from encoding genes after de-redundancy with a threshold of 95% sequence similarity by CD-HIT (94). The methods for calculating the gene abundance were according to Xue et al. (95). Briefly, the non-redundant gene catalogs were mapped against the clean reads of each metagenomic sample by BWA-MEM (version 0.7.17) using default parameters (96), and the average coverage of each gene was determined by BBMap (version 38.96) (97). To calculate the relative abundance, the average coverage of each gene was divided by the sum of the average coverage of all genes present in each metagenomic sample. Taxonomic assignment of each gene was performed with MEGAN (version 6.21.7) (98). Function assignment of each gene was performed by Diamond (version 0.8.23.85) using the BLASTP search with the e-value threshold of 1e-10 against the KEGG database (version 89), and only the best hit was retained (99, 100). Microbial sulfur metabolic genes were identified by KEGG serial number (Table S4).

### Genome binning and annotation

Contigs from each sample were used for metagenome binning with MetaWRAP integrated with MaxBin2 (model 2.2.4), metaBAT2 (model 2.12.1), and CONCOCT (model 0.4.0) (101 – 103). MAG sets were consolidated into a robust bin set with the Bin_refinement module and further reassembled by the Reassemble_bins module in MetaWRAP (104). The MAGs with completeness >50% and contamination <10%, which were evaluated by CheckM (105), were chosen for further analyses. dRep was used to remove replicated MAGs (106). The taxonomy assignment of each MAG was conducted by GTDB-Tk (version 0.3.2) (Table S5) (107). The relative abundance of MAGs in each sample of the SYBH was calculated using Anvi'o (version 6.2) and MicrobeCensus (108) and was illustrated using the Zero-to-One method at each MAG scale using TBtools (109). For each MAG in all the samples, the maximal relative abundance was set to one, and its scaled abundance in other samples was obtained by dividing the relative abundance by the maximal relative abundance. Gene calling and primary gene annotation of MAGs were conducted by Prokka (110). The gene function prediction of each MAG was performed using the KEGG Automatic Annotation Server, with the homology search using GhostX and the assignment method using single-directional best hit(111). The completeness of sulfur metabolic pathways in MAGs was determined by KEGG-Decoder (112).

### Phylogenetic analyses

A total of 57 nonredundant reference genomes of KSB1 (JdFR-76) and AABM5-125-24 were downloaded from GTDB (Table S6). A phylogenomic tree of the genomes from KSB1 (JdFR-76) and AABM5-125-24 was constructed based on the core single-copy sequences detected by OrthoFinder (113). Briefly, those single-copy sequences were aligned with MAFFT, trimmed with TrimAl and then submitted to IQ-TREE (version 1.6.1) for tree construction (114, 115). For the *dsrA* gene, after multiple sequence alignment by MAFFT (version 7) and trimming by TrimAl (version 1.2) (114, 115), its phylogenetic tree was constructed based on the maximum likelihood method using IQ-TREE (version 1.6.1) (116), and the oxidized and reduced types were determined using Blast against the DsrAB database constructed by Müller et al. (117).

## Data Availability

The datasets that support the findings of this study were deposited in the NCBI database under the project number PRJNA958612 (16S rRNA genes/transcripts sequencing data) and PRJNA958613 (metagenomic sequencing data).

## SUPPLEMENTAL MATERIAL

Supplemental file 1 spectrum.01149-23-s0001.docx Supplementary

ASM does not own the copyrights to Supplemental Material that may be linked to, or accessed through, an article. The authors have granted ASM a non-exclusive, world-wide license to publish the Supplemental Material files. Please contact the corresponding author directly for reuse.
