# Supplementary material for: Microbial communities related to the sulfur cycle in the Sansha Yongle Blue Hole
Source: Microbiol Spectr. 2023 Aug 25;11(5):e01149-23. doi: 10.1128/spectrum.01149-23 (PMC10580873; doi:10.1128/spectrum.01149-23)
Supplement: Supplemental file 1 — Supplementary [file spectrum.01149-23-s0001.docx]

**Supplementary material for：**

**Microbial communities related to the sulfur cycle in Sansha Yongle Blue Hole**

Kai Sun**^1^**^†^, Min Yu**^1,2,6^**^†^, Xiao-Yu Zhu**^1^**, Chun-Xu Xue**^1^**, Yunhui Zhang**^1,2,6^**, Xing Chen**^1^**, Peng Yao^2,3^, Lin Chen^2,3^, Liang Fu^4^, Zuosheng Yang^5^, Xiao-Hua Zhang**^1,2,6*^**

^1^Frontiers Science Center for Deep Ocean Multispheres and Earth System, and College of Marine Life Sciences, Ocean University of China, Qingdao 266003, China

^2^Laboratory for Marine Ecology and Environmental Science, Laoshan Laboratory, Qingdao 266237, China

^3^Key Laboratory of Marine Chemistry Theory and Technology, Ministry of Education, Ocean University of China, Qingdao 266071, China

^4^Sansha Track Ocean Coral Reef Conservation Research Institute, Sansha 573199, China

^5^College of Marine Geosciences, Ocean University of China, Qingdao, China

^6^Institute of Evolution & Marine Biodiversity, Ocean University of China, Qingdao 266003, China

**TABLE S1. Concentrations of different environmental parameters along the depths at the SYBH**

| Depth (m) | DOC (μmol/L) | NH_4_^+^ (μmol/L) | NO_3_^-^+NO_2_^-^ (μmol/L) | PO_4_^3-^ (μmol/L) | Salinity | Temp (^o^C) | DO (μmol/L) | H_2_S (μmol/L) | SO_4_^2-^ (μmol/L) |
| --- | --- | --- | --- | --- | --- | --- | --- | --- | --- |
| 0 | 90.75 | 1.37 | 1.25 | 0.02 | 33.65 | 28.66 | 191.98 | 0.00 | 29.45 |
| 10 | 73 | 0.81 | 0.01 | 0.12 | 33.67 | 28.22 | 121.53 | 0.00 | 29.54 |
| 20 | 70.98333 | 0.72 | 0.01 | 0.02 | 33.67 | 28.18 | 117.97 | 0.00 | 29.30 |
| 30 | 73.36667 | 0.65 | 0.01 | 0.03 | 33.68 | 28.17 | 119.75 | 0.00 | 28.15 |
| 40 | 45.075 | 0.37 | 0.01 | 0.03 | 33.68 | 28.14 | 113.83 | 0.00 | 27.87 |
| 50 | 43.76667 | 0.56 | 6.52 | 0.64 | 33.75 | 26.52 | 78.00 | 0.00 | 27.90 |
| 60 | 38.71667 | 0.46 | 9.5 | 0.95 | 33.87 | 24.40 | 63.16 | 0.00 | 28.26 |
| 65 | 21.79449 | 0.59 | 10.53 | 0.73 | 33.90 | 24.05 | 40.09 | 0.00 | 27.70 |
| 70 | 19.48615 | 0.48 | 10.13 | 0.69 | 33.98 | 23.42 | 36.73 | 0.00 | 27.97 |
| 75 | 21.11949 | 1.25 | 9.47 | 0.68 | 34.03 | 23.00 | 31.89 | 0.00 | 28.20 |
| 80 | 18.60282 | 0.46 | 10.59 | 0.8 | 34.11 | 22.40 | 24.25 | 0.00 | 27.99 |
| 85 | 21.62782 | 0.41 | 9.48 | 0.6 | 34.16 | 21.79 | 20.19 | 0.00 | 27.32 |
| 90 | 17.66949 | 0.48 | 2.71 | 0.59 | 34.24 | 20.83 | 12.25 | 0.01 | 28.07 |
| 95 | 10.74032 | 5.49 | 0.01 | 1.25 | 34.31 | 19.96 | 13.36 | 0.04 | 28.84 |
| 100 | 17.63615 | 11.18 | 0.01 | 1.34 | 34.35 | 19.26 | 8.06 | 2.51 | 27.87 |
| 105 | 15.06949 | 11.23 | 0.01 | 0.7 | 34.38 | 18.80 | 0.00 | 3.05 | 28.83 |
| 110 | 14.91949 | 15.95 | 0.01 | 1.24 | 34.41 | 18.37 | 0.00 | 4.22 | 27.20 |
| 115 | 15.38615 | 20.28 | 0.01 | 1.52 | 34.43 | 17.99 | 0.00 | 13.92 | 29.43 |
| 120 | 18.62782 | 21.68 | 0.01 | 2.01 | 34.45 | 17.88 | 0.00 | 22.07 | 28.18 |
| 125 | 11.81532 | 22.17 | 0.01 | 2.18 | 34.45 | 17.85 | 0.00 | 23.18 | 28.00 |
| 130 | 12.56615 | 19.63 | 0.01 | 2.06 | 34.44 | 17.71 | 0.00 | 20.52 | 28.62 |
| 135 | 14.96949 | 27.13 | 0.01 | 2.48 | 34.44 | 17.37 | 0.00 | 38.22 | 26.95 |
| 140 | 19.84449 | 40.36 | 0.01 | 3.33 | 34.44 | 16.55 | 0.00 | 94.24 | 28.05 |
| 145 | 22.79449 | 66.08 | 0.01 | 4.32 | 34.48 | 15.68 | 0.00 | 214.49 | 27.99 |
| 150 | 29.24449 | 85.14 | 0.01 | 5.24 | 34.48 | 15.56 | 0.00 | 249.50 | 26.70 |
| 160 | 18.33333 | 72.83 | 0.01 | 4.51 | 34.48 | 15.50 | 0.00 | 243.90 | 26.39 |
| 170 | 22.26667 | 82.38 | 0.01 | 4.66 | 34.48 | 15.49 | 0.00 | 253.50 | 26.29 |
| 180 | 27.41667 | 86.08 | 0.01 | 5.22 | 34.48 | 15.48 | 0.00 | 246.30 | 27.14 |
| 190 | 19.975 | 80.89 | 0.01 | 4.61 | 34.48 | 15.43 | 0.00 | 251.10 | 25.99 |

**TABLE S2. Numbers of sequences and bases in different 16S rRNA genes and transcripts sampling groups**

| Sample | Sequence number | Base number |
| --- | --- | --- |
| F0_D | 49039407 | 12408239967 |
| F0_R | 4067571799 | 1.02917E+12 |
| F10_D | 42256472 | 10691928784 |
| F10_R | 3759135635 | 9.51126E+11 |
| F20_D | 4598014 | 1163407854 |
| F20_R | 707450712 | 1.78997E+11 |
| F30_D | 34188910 | 8650600028 |
| F30_R | 3348846085 | 8.47315E+11 |
| F40_D | 3395139 | 859006353 |
| F40_R | 204482098 | 51737463096 |
| F50_D | 32336967 | 8182003133 |
| F50_R | 3246401738 | 8.21395E+11 |
| F60_D | 6530791 | 1652505055 |
| F60_R | 1110990274 | 2.811E+11 |
| F65_D | 5444085 | 1377511610 |
| F65_R | 909112782 | 2.30021E+11 |
| F70_D | 44446715 | 11246126880 |
| F70_R | 3861875404 | 9.77121E+11 |
| F75_D | 40139564 | 10156288041 |
| F75_R | 3656466921 | 9.25149E+11 |
| F80_D | 75239705 | 19037157158 |
| F80_R | 606717454 | 1.5351E+11 |
| F85_D | 3957471 | 1001312442 |
| F85_R | 506057416 | 1.28041E+11 |
| F90_D | 16856816 | 4265190336 |
| F90_R | 2225809582 | 5.63168E+11 |
| F95_D | 18170472 | 4597552788 |
| F95_R | 2327546260 | 5.88909E+11 |
| F100_D | 100327405 | 25384537525 |
| F100_R | 5930892890 | 1.50062E+12 |
| F105_D | 93691723 | 23705647788 |
| F105_R | 5722804218 | 1.44797E+12 |
| F110_D | 22431344 | 5675618119 |
| F110_R | 2633189804 | 6.66242E+11 |
| F115_D | 19543744 | 4945013519 |
| F115_R | 2429357645 | 6.14669E+11 |
| F120_D | 61487593 | 15557811977 |
| F120_R | 4582993336 | 1.15958E+12 |
| F125_D | 25525639 | 6458548992 |
| F125_R | 2837312806 | 7.17889E+11 |
| F130_D | 46707969 | 11818303808 |
| F130_R | 3964687893 | 1.00313E+12 |
| F135_D | 23941665 | 6057761597 |
| F135_R | 2735214623 | 6.92056E+11 |
| F140_D | 56323873 | 14251377924 |
| F140_R | 4376628422 | 1.10736E+12 |
| F145_D | 64142239 | 16229450399 |
| F145_R | 4686283187 | 1.18571E+12 |
| F150_D | 7178618 | 1816444428 |
| F150_R | 1212024960 | 3.06663E+11 |
| F160_D | 90463402 | 22888850069 |
| F160_R | 5618860116 | 1.42167E+12 |
| F170_D | 14441070 | 3654018938 |
| F170_R | 2022530773 | 5.11735E+11 |
| F180_D | 3705023 | 937423755 |
| F180_R | 405468357 | 1.0259E+11 |
| F190_D | 28830750 | 7294832845 |
| F190_R | 3041713312 | 7.69606E+11 |
| P0_D | 27161698 | 6872515460 |
| P0_R | 2939475767 | 7.43738E+11 |
| P10_D | 15614420 | 3950863310 |
| P10_R | 2124133578 | 5.37442E+11 |
| P20_D | 87283057 | 22084195518 |
| P20_R | 5514986991 | 1.39539E+12 |
| P30_D | 20969425 | 5305727529 |
| P30_R | 2531237912 | 6.40447E+11 |
| P40_D | 96988784 | 24539833457 |
| P40_R | 5826812099 | 1.47428E+12 |
| P50_D | 72383591 | 18314546547 |
| P50_R | 4996569038 | 1.26422E+12 |
| P60_D | 51402790 | 13006242897 |
| P60_R | 4170517283 | 1.05521E+12 |
| P65_D | 58882514 | 14898720164 |
| P65_R | 4479778086 | 1.13346E+12 |
| P70_D | 10360208 | 2621516077 |
| P70_R | 1616782688 | 4.09074E+11 |
| P75_R | 1414290090 | 3.5784E+11 |
| P80_D | 4244759 | 1074014408 |
| P80_R | 5100113614 | 1.29042E+12 |
| P85_D | 81143667 | 20530890077 |
| P85_R | 5307407064 | 1.34287E+12 |
| P90_D | 84176112 | 21298118803 |
| P90_R | 5411164259 | 1.36912E+12 |
| P95_D | 13337166 | 3374714765 |
| P95_R | 1920991001 | 4.86044E+11 |
| P100_D | 78161299 | 19776333327 |
| P100_R | 5203724290 | 1.31663E+12 |
| P105_D | 69591900 | 17608239124 |
| P105_R | 4893071226 | 1.23803E+12 |
| P110_D | 8643945 | 2187252061 |
| P110_R | 1414257401 | 3.57831E+11 |
| P115_D | 11292031 | 2857276348 |
| P115_R | 1718118658 | 4.34713E+11 |
| P120_D | 66834819 | 16910684802 |
| P120_R | 4789643235 | 1.21186E+12 |
| P125_D | 53826222 | 13619417543 |
| P125_R | 4273537327 | 1.08128E+12 |
| P130_D | 9458885 | 2393472068 |
| P130_R | 1515505404 | 3.83449E+11 |
| P135_D | 7883054 | 1994706829 |
| P135_R | 1313110845 | 3.32239E+11 |
| P140_D | 36107973 | 9136178989 |
| P140_R | 3451315870 | 8.73242E+11 |
| P145_D | 38093374 | 9638541606 |
| P145_R | 3553856055 | 8.99186E+11 |
| P150_D | 4987194 | 1261892345 |
| P150_R | 808249709 | 2.04501E+11 |
| P160_D | 3526319 | 892198470 |
| P160_R | 304949444 | 77157412242 |
| P170_D | 12279681 | 3107161177 |
| P170_R | 1819524351 | 4.60371E+11 |
| P180_D | 30546407 | 7728939657 |
| P180_R | 3144022305 | 7.95491E+11 |
| P190_D | 5952891 | 1506266033 |
| P190_R | 1010028204 | 2.55554E+11 |

**TABLE S3. Information of metagenomes used in this study.**

| Sample | Raw base | Clean base | UtilizationRatio (%) | Contig Number | Assembly Length (bp) | N50 (bp) | N90 (bp) | Max (bp) | Min (bp) | Average Size (bp) | Gene number |
| --- | --- | --- | --- | --- | --- | --- | --- | --- | --- | --- | --- |
| F0 | 14460946500 | 14112812100 | 97.59 | 585054 | 378501332 | 683 | 353 | 91239 | 300 | 646 | 564371 |
| F30 | 13705133700 | 13043386500 | 95.17 | 586530 | 378945475 | 679 | 352 | 217802 | 300 | 646 | 675130 |
| F50 | 14460946500 | 14198349900 | 98.18 | 499679 | 331906201 | 710 | 356 | 53500 | 300 | 664 | 560162 |
| F90 | 14460946500 | 14208683700 | 98.26 | 309289 | 338378958 | 1545 | 449 | 362831 | 300 | 1094 | 384943 |
| F120 | 14460946500 | 14132852400 | 97.73 | 430875 | 482651824 | 1608 | 461 | 434417 | 300 | 1120 | 689924 |
| F140 | 13605166800 | 13226257500 | 97.21 | 447105 | 506634679 | 1648 | 462 | 361914 | 300 | 1133 | 733008 |
| F170 | 14460946500 | 14237255100 | 98.45 | 567159 | 569087225 | 1274 | 441 | 293296 | 300 | 1003 | 653057 |
| P0 | 14986799100 | 14196534300 | 94.73 | 251271 | 153532242 | 632 | 347 | 56372 | 300 | 611 | 400775 |
| P30 | 14390338800 | 13798765800 | 95.89 | 406924 | 254151373 | 652 | 350 | 57657 | 300 | 624 | 411369 |
| P50 | 14342690700 | 13662367500 | 95.26 | 165659 | 89793586 | 537 | 330 | 96117 | 300 | 542 | 272731 |
| P90 | 14460946500 | 14163247500 | 97.94 | 356584 | 406653051 | 1791 | 445 | 224518 | 300 | 1140 | 440011 |
| P120 | 14460946500 | 14140300800 | 97.78 | 315820 | 406815898 | 2284 | 488 | 434417 | 300 | 1288 | 681587 |
| P140 | 12881156700 | 12555987900 | 97.48 | 419441 | 503744295 | 1880 | 474 | 401524 | 300 | 1200 | 619612 |
| P170 | 13166838300 | 12805847100 | 97.26 | 457798 | 504677971 | 1566 | 456 | 265205 | 300 | 1102 | 657405 |

**TABLE S4. Summary of sulfur cycling genes with KEGG number**

| Gene | KO | Annotation |
| --- | --- | --- |
| *sqr* | K17218 | Sulfide:quinone oxidoreductase |
| *fccA* | K17230 | Cytochrome subunit of sulfide dehydrogenase |
| *fccB* | K17229 | Sulfide dehydrogenase [flavocytochrome c] flavoprotein chain |
| *soxA* | K17222 | L-cysteine S-thiosulfotransferase |
| *soxB* | K17224 | S-sulfosulfanyl-L-cysteine sulfohydrolase |
| *soxC* | K17225 | Sulfane dehydrogenase subunit SoxC |
| *soxD* | K22622 | S-disulfanyl-L-cysteine oxidoreductase SoxD |
| *soxX* | K17223 | L-cysteine S-thiosulfotransferase |
| *soxY* | K17226 | Sulfur-oxidizing protein SoxY |
| *soxZ* | K17227 | Sulfur-oxidizing protein SoxZ |
| *soeA* | K21307 | Sulfite dehydrogenase (quinone) subunit SoeA |
| *soeB* | K21308 | Sulfite dehydrogenase (quinone) subunit SoeB |
| *soeC* | K21309 | Sulfite dehydrogenase (quinone) subunit SoeC |
| *sat* | K00958 | Sulfate adenylyltransferase |
| *aprA* | K00394 | Adenylylsulfate reductase, subunit A |
| *aprB* | K00395 | Adenylylsulfate reductase, subunit B |
| *dsrA* | K11180 | Dissimilatory sulfite reductase alpha subunit |
| *dsrB* | K11181 | Dissimilatory sulfite reductase beta subunit |
| *hydA* | K17993 | Sulfhydrogenase subunit alpha |
| *hydB* | K17996 | Sulfhydrogenase subunit beta (sulfur reductase) |
| *hydD* | K17994 | Sulfhydrogenase subunit delta |
| *hydG* | K17995 | Sulfhydrogenase subunit gamma (sulfur reductase) |
| *phsA* | K08352 | Thiosulfate reductase |
| *phsB* | K08353 | Thiosulfate reductase electron transport protein |
| *phsC* | K08354 | Thiosulfate reductase cytochrome b subunit |

**TABLE S5. Information of 108 MAGs retrieved from the SYBH**

| GenomeID | Completeness | Contamination | Size | GC | N50 | GTDB classification |
| --- | --- | --- | --- | --- | --- | --- |
| MAG10-F30 | 99 | 0.238 | 3271994 | 0.524 | 80515 | d__Bacteria;p__Proteobacteria;c__Gammaproteobacteria;o__Burkholderiales;f__Burkholderiaceae;g__Limnobacter;s__Limnobacter sp002954425 |
| MAG11-F30 | 85.31 | 1.131 | 1780545 | 0.537 | 3219 | d__Bacteria;p__Proteobacteria;c__Alphaproteobacteria;o__Puniceispirillales;f__Puniceispirillaceae;g__HIMB100;s__ |
| MAG125-F120 | 98.8 | 0 | 1954067 | 0.412 | 30096 | d__Bacteria;p__Marinisomatota;c__Marinisomatia;o__Marinisomatales;f__UBA8229;g__;s__ |
| MAG126-F120 | 98.8 | 0.297 | 3917139 | 0.445 | 34395 | d__Bacteria;p__Desulfobacterota;c__Desulfobulbia;o__Desulfobulbales;f__Desulfocapsaceae;g__;s__ |
| MAG128-F120 | 97.8 | 0.099 | 3610288 | 0.697 | 17818 | d__Bacteria;p__Krumholzibacteriota;c__Krumholzibacteria;o__LZORAL124-64-63;f__;g__;s__ |
| MAG131-F120 | 97.74 | 0.967 | 4681308 | 0.531 | 18745 | d__Bacteria;p__Desulfobacterota;c__Desulfobacteria;o__Desulfobacterales;f__;g__;s__ |
| MAG138-F120 | 94.38 | 0 | 1085377 | 0.343 | 269511 | d__Bacteria;p__Elusimicrobiota;c__Elusimicrobia;o__Elusimicrobiales;f__Elusimicrobiaceae;g__;s__ |
| MAG139-F120 | 93.45 | 2.803 | 2881464 | 0.505 | 804319 | d__Archaea;p__Thermoproteota;c__Bathyarchaeia;o__TCS64;f__TCS64;g__RBG-16-57-9;s__ |
| MAG141-F120 | 92.43 | 2.38 | 4647507 | 0.438 | 27303 | d__Bacteria;p__Bacteroidota;c__Bacteroidia;o__Bacteroidales;f__GCA-2748055;g__;s__ |
| MAG144-F120 | 88.7 | 2.346 | 1832899 | 0.421 | 29711 | d__Bacteria;p__Omnitrophota;c__Koll11;o__UBA1560;f__SKK-01;g__;s__ |
| MAG145-F120 | 87.42 | 0 | 1256062 | 0.395 | 111983 | d__Bacteria;p__Omnitrophota;c__Koll11;o__4484-171;f__4484-171;g__;s__ |
| MAG146-F120 | 87.02 | 2.903 | 6747279 | 0.474 | 8692 | d__Bacteria;p__Desulfobacterota;c__Desulfobacteria;o__Desulfobacterales;f__;g__;s__ |
| MAG147-F120 | 86.35 | 0.967 | 3957104 | 0.538 | 10545 | d__Bacteria;p__Desulfobacterota;c__Desulfobacteria;o__Desulfobacterales;f__;g__;s__ |
| MAG148-F120 | 86.13 | 0 | 1323136 | 0.392 | 111643 | d__Bacteria;p__Omnitrophota;c__Koll11;o__4484-171;f__4484-171;g__;s__ |
| MAG15-F50 | 80.83 | 0.537 | 1435276 | 0.36 | 6593 | d__Bacteria;p__Bacteroidota;c__Bacteroidia;o__Flavobacteriales;f__UBA7430;g__UBA6772;s__ |
| MAG151-F120 | 81.57 | 2.311 | 5424257 | 0.488 | 5918 | d__Bacteria;p__Desulfobacterota;c__Desulfobacteria;o__Desulfatiglandales;f__;g__;s__ |
| MAG152-F120 | 80.64 | 3.529 | 14338817 | 0.589 | 7914 | d__Bacteria;p__Planctomycetota;c__Planctomycetes;o__Pirellulales;f__Pirellulaceae;g__;s__ |
| MAG173-P120 | 97.8 | 0 | 2580610 | 0.653 | 99447 | d__Bacteria;p__Eisenbacteria;c__;o__;f__;g__;s__ |
| MAG175-P120 | 97.72 | 2.272 | 3860720 | 0.47 | 142697 | d__Bacteria;p__Planctomycetota;c__Phycisphaerae;o__Sedimentisphaerales;f__Anaerohalophaeraceae;g__4572-13;s__ |
| MAG176-P120 | 97.38 | 2.539 | 5166748 | 0.494 | 46334 | d__Bacteria;p__Bacteroidota;c__Bacteroidia;o__Bacteroidales;f__GCA-2748055;g__GCA-2748055;s__ |
| MAG178-P120 | 96.52 | 2.272 | 6302166 | 0.61 | 102505 | d__Bacteria;p__Planctomycetota;c__UBA1135;o__B15-G4;f__;g__;s__ |
| MAG179-P120 | 96.45 | 1.612 | 6171394 | 0.48 | 42925 | d__Bacteria;p__Desulfobacterota;c__Desulfobacteria;o__Desulfobacterales;f__B30-G6;g__B30-G6;s__ |
| MAG180-P120 | 95.69 | 5.913 | 8021145 | 0.587 | 44437 | d__Bacteria;p__Planctomycetota;c__UBA8108;o__UBA8890;f__UBA8898;g__;s__ |
| MAG181-P120 | 95.45 | 7.272 | 2691268 | 0.461 | 407236 | d__Bacteria;p__Chloroflexota;c__Anaerolineae;o__Anaerolineales;f__Anaerolineaceae;g__Pelolinea;s__ |
| MAG185-P120 | 93.47 | 0.8 | 2773886 | 0.548 | 35165 | d__Archaea;p__Thermoplasmatota;c__Poseidoniia;o__MGIII;f__;g__;s__ |
| MAG187-P120 | 92.07 | 0.649 | 4668698 | 0.506 | 12411 | d__Bacteria;p__Abyssubacteria;c__SURF-5;o__SURF-5;f__;g__;s__ |
| MAG188-P120 | 91.81 | 3.818 | 5155863 | 0.497 | 16397 | d__Bacteria;p__Chloroflexota;c__Anaerolineae;o__Anaerolineales;f__UBA11858;g__J130;s__ |
| MAG189-P120 | 91.36 | 2.727 | 6172761 | 0.495 | 47490 | d__Bacteria;p__Chloroflexota;c__Anaerolineae;o__Anaerolineales;f__UBA11858;g__J130;s__ |
| MAG191-P120 | 89 | 0.701 | 1733105 | 0.562 | 9649 | d__Bacteria;p__Proteobacteria;c__Gammaproteobacteria;o__GCF-002020875;f__GCF-002020875;g__;s__ |
| MAG197-P120 | 82.95 | 4.879 | 5128126 | 0.622 | 7329 | d__Bacteria;p__Planctomycetota;c__UBA1135;o__B15-G4;f__;g__;s__ |
| MAG199-P120 | 77.75 | 6.14 | 7307310 | 0.689 | 3898 | d__Bacteria;p__Myxococcota;c__UBA796;o__UBA9615;f__;g__;s__ |
| MAG212-F140 | 98.92 | 1.075 | 4123170 | 0.416 | 147813 | d__Bacteria;p__Bacteroidota;c__Bacteroidia;o__Bacteroidales;f__GCA-2711565;g__;s__ |
| MAG214-F140 | 98.8 | 1.587 | 4795626 | 0.476 | 358065 | d__Bacteria;p__Bacteroidota;c__Bacteroidia;o__Bacteroidales;f__GCA-2748055;g__GCA-2748055;s__ |
| MAG218-F140 | 97.35 | 0.793 | 5284863 | 0.486 | 37923 | d__Bacteria;p__Bacteroidota;c__Bacteroidia;o__Bacteroidales;f__SM23-62;g__SM23-62;s__ |
| MAG219-F140 | 97.31 | 2.419 | 3588858 | 0.361 | 225912 | d__Bacteria;p__Bacteroidota;c__Bacteroidia;o__Bacteroidales;f__4484-276;g__;s__ |
| MAG22-P50 | 90.86 | 0.017 | 1617907 | 0.419 | 22508 | d__Bacteria;p__Bacteroidota;c__Bacteroidia;o__Flavobacteriales;f__UA16;g__UBA974;s__ |
| MAG222-F140 | 95.43 | 1.098 | 3516141 | 0.478 | 9990 | d__Bacteria;p__Marinisomatota;c__Marinisomatia;o__Marinisomatales;f__TCS56;g__;s__ |
| MAG228-F140 | 91.2 | 2.336 | 1395271 | 0.493 | 26799 | d__Archaea;p__Altarchaeota;c__Altarchaeia;o__IMC4;f__QMZM01;g__;s__ |
| MAG230-F140 | 88.38 | 0 | 3511434 | 0.478 | 10088 | d__Bacteria;p__Desulfobacterota;c__Desulfobacteria;o__Desulfobacterales;f__Desulfosarcinaceae;g__;s__ |
| MAG235-F140 | 86.17 | 2.735 | 2069333 | 0.445 | 9694 | d__Bacteria;p__Nitrospinota_B;c__2-12-FULL-45-22;o__;f__;g__;s__ |
| MAG236-F140 | 86.12 | 0.99 | 1193278 | 0.486 | 4866 | d__Bacteria;p__Chloroflexota;c__Dehalococcoidia;o__Dehalococcoidales;f__UBA5760;g__;s__ |
| MAG237-F140 | 85.98 | 1.473 | 1559744 | 0.314 | 13271 | d__Archaea;p__Nanoarchaeota;c__Nanoarchaeia;o__Woesearchaeales;f__UBA10107;g__;s__ |
| MAG238-F140 | 85.86 | 0.99 | 1248229 | 0.577 | 8568 | d__Bacteria;p__Chloroflexota;c__Dehalococcoidia;o__Dehalococcoidales;f__UBA5760;g__UBA5760;s__ |
| MAG239-F140 | 85.64 | 0 | 1440592 | 0.447 | 37346 | d__Bacteria;p__Patescibacteria;c__Gracilibacteria;o__UBA1369;f__UBA12473;g__;s__ |
| MAG240-F140 | 85.16 | 0 | 2495289 | 0.392 | 8875 | d__Bacteria;p__Marinisomatota;c__Marinisomatia;o__Marinisomatales;f__SCKK01;g__;s__ |
| MAG243-F140 | 82.67 | 0 | 1295655 | 0.487 | 36396 | d__Bacteria;p__Patescibacteria;c__Gracilibacteria;o__UBA1369;f__UBA12473;g__;s__ |
| MAG246-F140 | 79.14 | 4.574 | 7129232 | 0.441 | 5740 | d__Bacteria;p__Poribacteria;c__;o__;f__;g__;s__ |
| MAG247-F140 | 79.09 | 0 | 775801 | 0.343 | 74192 | d__Bacteria;p__Patescibacteria;c__Paceibacteria;o__Paceibacterales;f__CG1-02-41-26;g__;s__ |
| MAG26-F90 | 94.15 | 1.741 | 4250933 | 0.526 | 39162 | d__Bacteria;p__Proteobacteria;c__Alphaproteobacteria;o__Rhodospirillales;f__2-02-FULL-58-16_A;g__;s__ |
| MAG262-P140 | 98.9 | 0 | 2994114 | 0.385 | 115872 | d__Bacteria;p__Cloacimonadota;c__Cloacimonadia;o__JGIOTU-2;f__JGIOTU-2;g__;s__ |
| MAG263-P140 | 98.86 | 4.545 | 13135526 | 0.555 | 28588 | d__Bacteria;p__Planctomycetota;c__Phycisphaerae;o__Sedimentisphaerales;f__Anaerohalophaeraceae;g__UBA2266;s__ |
| MAG273-P140 | 94.5 | 0.099 | 3111780 | 0.631 | 19629 | d__Bacteria;p__Krumholzibacteriota;c__Krumholzibacteria;o__;f__;g__;s__ |
| MAG279-P140 | 89.32 | 1.727 | 4805060 | 0.644 | 41440 | d__Bacteria;p__Myxococcota;c__UBA9042;o__PWKZ01;f__;g__;s__ |
| MAG28-F90 | 93.1 | 2.24 | 2452133 | 0.361 | 68775 | d__Bacteria;p__Bacteroidota;c__Bacteroidia;o__Flavobacteriales;f__UBA7430;g__;s__ |
| MAG280-P140 | 88.33 | 4.727 | 6578664 | 0.59 | 14353 | d__Bacteria;p__Chloroflexota;c__Anaerolineae;o__UBA7937;f__E26-bin7;g__;s__ |
| MAG283-P140 | 86.6 | 1.123 | 1137801 | 0.397 | 72870 | d__Bacteria;p__Patescibacteria;c__Paceibacteria_A;o__Moranbacterales;f__UBA2193;g__;s__ |
| MAG287-P140 | 79.35 | 2.974 | 4270969 | 0.451 | 7121 | d__Bacteria;p__Desulfobacterota;c__Desulfobacteria;o__Desulfobacterales;f__Desulfobacteraceae;g__;s__ |
| MAG29-F90 | 92.62 | 2.15 | 2431876 | 0.35 | 25362 | d__Bacteria;p__Bacteroidota;c__Bacteroidia;o__Flavobacteriales;f__UBA7430;g__UBA6772;s__ |
| MAG30-F90 | 92.31 | 0.898 | 2004091 | 0.606 | 22322 | d__Bacteria;p__Proteobacteria;c__Alphaproteobacteria;o__UBA11136;f__UBA11136;g__UBA11136;s__ |
| MAG306-F170 | 98.92 | 2.15 | 5455847 | 0.33 | 35320 | d__Bacteria;p__Bacteroidota;c__Bacteroidia;o__Bacteroidales;f__GCA-2711565;g__;s__ |
| MAG31-F90 | 92.28 | 4.27 | 2871406 | 0.578 | 11809 | d__Bacteria;p__Proteobacteria;c__Alphaproteobacteria;o__Rhodospirillales;f__Casp-alpha2;g__Casp-alpha2;s__ |
| MAG310-F170 | 97.73 | 1.098 | 4092588 | 0.503 | 37884 | d__Bacteria;p__AABM5-125-24;c__B3-LCP;o__;f__;g__;s__ |
| MAG311-F170 | 97.73 | 0 | 3311321 | 0.473 | 44685 | d__Bacteria;p__AABM5-125-24;c__AABM5-125-24;o__AABM5-125-24;f__AABM5-125-24;g__;s__ |
| MAG315-F170 | 91.83 | 4.919 | 16119694 | 0.586 | 20022 | d__Bacteria;p__Planctomycetota;c__Planctomycetes;o__Pirellulales;f__Pirellulaceae;g__;s__ |
| MAG316-F170 | 90.32 | 4.7 | 4934462 | 0.454 | 96649 | d__Bacteria;p__Acidobacteriota;c__Aminicenantia;o__Aminicenantales;f__Aminicenantaceae;g__;s__ |
| MAG318-F170 | 88.69 | 0 | 1248004 | 0.459 | 32106 | d__Bacteria;p__Omnitrophota;c__Koll11;o__4484-171;f__4484-171;g__;s__ |
| MAG32-F90 | 90.2 | 2.072 | 3184624 | 0.542 | 15453 | d__Bacteria;p__Proteobacteria;c__Gammaproteobacteria;o__Pseudomonadales;f__HTCC2089;g__UBA9659;s__ |
| MAG322-F170 | 83.64 | 0.934 | 1323775 | 0.327 | 114904 | d__Archaea;p__Nanoarchaeota;c__Nanoarchaeia;o__Woesearchaeales;f__GW2011-AR9;g__GCA-2688265;s__ |
| MAG323-F170 | 81.2 | 0.8 | 3134535 | 0.453 | 7856 | d__Archaea;p__Thermoplasmatota;c__;o__;f__;g__;s__ |
| MAG324-F170 | 80.1 | 2.15 | 1159813 | 0.414 | 37273 | d__Bacteria;p__Omnitrophota;c__Koll11;o__UBA10183;f__UBA10183;g__;s__ |
| MAG33-F90 | 88.79 | 2.411 | 3959370 | 0.628 | 5029 | d__Bacteria;p__Verrucomicrobiota;c__Verrucomicrobiae;o__Opitutales;f__MB11C04;g__GCA-2730975;s__ |
| MAG330-F170 | 75.54 | 1.006 | 1561861 | 0.348 | 9151 | d__Archaea;p__Nanoarchaeota;c__Nanoarchaeia;o__Woesearchaeales;f__UBA11716;g__;s__ |
| MAG34-F90 | 88.7 | 0.41 | 1472178 | 0.351 | 88131 | d__Bacteria;p__Proteobacteria;c__Gammaproteobacteria;o__UBA4486;f__UBA4486;g__UBA11869;s__UBA11869 sp002721165 |
| MAG35-F90 | 86.86 | 2.197 | 2326220 | 0.408 | 9445 | d__Bacteria;p__Marinisomatota;c__Marinisomatia;o__Marinisomatales;f__UBA1611;g__GCA-2722105;s__ |
| MAG355-P170 | 98.85 | 1.149 | 9244814 | 0.622 | 46745 | d__Bacteria;p__Planctomycetota;c__Planctomycetes;o__Pirellulales;f__UBA11386;g__;s__ |
| MAG357-P170 | 97.8 | 1.098 | 4076452 | 0.479 | 45374 | d__Bacteria;p__Marinisomatota;c__Marinisomatia;o__SCGC-AAA003-L08;f__;g__;s__ |
| MAG361-P170 | 95.69 | 0.537 | 5443962 | 0.387 | 27101 | d__Bacteria;p__Bacteroidota;c__Bacteroidia;o__Bacteroidales;f__4484-276;g__;s__ |
| MAG362-P170 | 92.94 | 1.075 | 1749235 | 0.407 | 81270 | d__Bacteria;p__Omnitrophota;c__Koll11;o__;f__;g__;s__ |
| MAG364-P170 | 89.74 | 1.29 | 5131534 | 0.563 | 10477 | d__Bacteria;p__Desulfobacterota;c__Desulfomonilia;o__Desulfomonilales;f__Desulfomonilaceae;g__;s__ |
| MAG371-P170 | 78.91 | 3.846 | 5764229 | 0.36 | 11866 | d__Bacteria;p__JdFR-76; c__JdFR-76;o__JdFR-76;f__JdFR-76;g__;s__ |
| MAG372-P170 | 77.77 | 0 | 2846704 | 0.486 | 6381 | d__Bacteria;p__Desulfobacterota;c__Desulfobacteria;o__Desulfobacterales;f__Desulfobacteraceae;g__Desulfobacter;s__ |
| MAG375-P170 | 75.76 | 0.681 | 5879916 | 0.58 | 7463 | d__Bacteria;p__Myxococcota;c__UBA9042;o__PWKZ01;f__;g__;s__ |
| MAG38-F90 | 84.21 | 0 | 2874835 | 0.556 | 16255 | d__Bacteria;p__Proteobacteria;c__Alphaproteobacteria;o__Rhodospirillales;f__2-02-FULL-58-16_A;g__;s__ |
| MAG40-F90 | 81.2 | 4.467 | 1264439 | 0.364 | 10751 | d__Bacteria;p__Proteobacteria;c__Gammaproteobacteria;o__SAR86;f__SAR86;g__AEGEAN-183;s__ |
| MAG42-F90 | 76.85 | 1.282 | 1771455 | 0.456 | 4583 | d__Bacteria;p__Nitrospinota;c__Nitrospinia;o__Nitrospinales;f__Nitrospinaceae;g__SCGCAAA288-L16;s__ |
| MAG43-F90 | 76.64 | 1.059 | 1375147 | 0.432 | 12208 | d__Bacteria;p__Verrucomicrobiota_A;c__Chlamydiia;o__2-12-FULL-49-11;f__2-12-FULL-49-11;g__;s__ |
| MAG44-F90 | 76.2 | 0.918 | 1370608 | 0.393 | 22329 | d__Bacteria;p__Proteobacteria;c__Gammaproteobacteria;o__UBA11654;f__UBA11654;g__UBA11654;s__ |
| MAG73-P90 | 99.32 | 2.732 | 4774996 | 0.516 | 66962 | d__Bacteria;p__Verrucomicrobiota;c__Verrucomicrobiae;o__Opitutales;f__Opitutaceae;g__UBA5691;s__ |
| MAG74-P90 | 98.75 | 1.176 | 7310081 | 0.571 | 27368 | d__Bacteria;p__Planctomycetota;c__Planctomycetes;o__Pirellulales;f__Pirellulaceae;g__;s__ |
| MAG75-P90 | 96.88 | 1.111 | 8862285 | 0.527 | 20904 | d__Bacteria;p__Planctomycetota;c__Planctomycetes;o__Planctomycetales;f__Planctomycetaceae;g__;s__ |
| MAG76-P90 | 96.42 | 0.549 | 2242346 | 0.49 | 24124 | d__Bacteria;p__Bacteroidota;c__Chlorobia;o__Chlorobiales;f__Chlorobiaceae;g__Chlorobium_A;s__ |
| MAG77-P90 | 96.4 | 0 | 5094511 | 0.582 | 64063 | d__Bacteria;p__Planctomycetota;c__Planctomycetes;o__Pirellulales;f__Pirellulaceae;g__SAT2750;s__ |
| MAG78-P90 | 95.16 | 1.173 | 2477615 | 0.591 | 291365 | d__Bacteria;p__Planctomycetota;c__UBA1135;o__UBA1135;f__UBA1135;g__GCA-2746235;s__ |
| MAG79-P90 | 91.72 | 1.712 | 4816906 | 0.578 | 36659 | d__Bacteria;p__Proteobacteria;c__Gammaproteobacteria;o__Pseudomonadales;f__HTCC2089;g__UBA4421;s__ |
| MAG80-P90 | 91.28 | 2.231 | 2166251 | 0.656 | 28794 | d__Bacteria;p__Actinobacteriota;c__Acidimicrobiia;o__Acidimicrobiales;f__MedAcidi-G1;g__UBA3125;s__UBA3125 sp002687745 |
| MAG81-P90 | 91.12 | 0 | 4745151 | 0.667 | 14552 | d__Bacteria;p__Myxococcota_A;c__UBA9160;o__UBA9160;f__SMWR01;g__;s__ |
| MAG83-P90 | 88.61 | 1.936 | 2115936 | 0.57 | 37736 | d__Bacteria;p__Proteobacteria;c__Gammaproteobacteria;o__GCA-2729495;f__GCA-2729495;g__GCA-2729495;s__ |
| MAG84-P90 | 87.56 | 1.263 | 1902705 | 0.404 | 6959 | d__Bacteria;p__Marinisomatota;c__Marinisomatia;o__Marinisomatales;f__TCS55;g__;s__ |
| MAG85-P90 | 87.22 | 0 | 1903240 | 0.365 | 110308 | d__Bacteria;p__Bdellovibrionota;c__Oligoflexia;o__Oligoflexales;f__Bog-1112;g__;s__ |
| MAG86-P90 | 85.6 | 2.741 | 6123331 | 0.626 | 5933 | d__Bacteria;p__Myxococcota;c__UBA796;o__UBA796;f__UBA796;g__;s__ |
| MAG87-P90 | 84.24 | 1.173 | 2062803 | 0.555 | 8730 | d__Bacteria;p__Planctomycetota;c__UBA1135;o__UBA1135;f__UBA1135;g__NORP165;s__ |
| MAG88-P90 | 83.82 | 0 | 1084271 | 0.402 | 28294 | d__Archaea;p__Thermoplasmatota;c__Poseidoniia;o__MGIII;f__CG-Epi1;g__CG-Epi1;s__ |
| MAG89-P90 | 83.06 | 0.537 | 4132483 | 0.7 | 4757 | d__Bacteria;p__Planctomycetota;c__UBA1135;o__UBA1135;f__GCA-002686595;g__;s__ |
| MAG90-P90 | 82.84 | 3.826 | 7890470 | 0.633 | 7256 | d__Bacteria;p__Planctomycetota;c__UBA8108;o__UBA8108;f__UBA8108;g__;s__ |
| MAG92-P90 | 81.06 | 0.8 | 1842600 | 0.467 | 36941 | d__Archaea;p__Thermoplasmatota;c__Poseidoniia;o__Poseidoniales;f__Poseidoniaceae;g__UBA226;s__ |
| MAG94-P90 | 79.1 | 0 | 2311661 | 0.479 | 5203 | d__Bacteria;p__Planctomycetota;c__UBA1135;o__UBA1135;f__UBA1135;g__NORP165;s__ |
| MAG95-P90 | 77.09 | 0 | 1443090 | 0.499 | 24612 | d__Archaea;p__Thermoplasmatota;c__Poseidoniia;o__Poseidoniales;f__Thalassarchaeaceae;g__Thalassarchaeum;s__ |
| MAG96-P90 | 75.48 | 0.387 | 3589262 | 0.675 | 4185 | d__Bacteria;p__Planctomycetota;c__GCA-002687715;o__GCA-002687715;f__GCA-002687715;g__GCA-2683135;s__ |

**TABLE S6. Information of reference MAGs of JdFR-76 and AABM5-125-4**

| GenomeID | Completeness | Contamination | Contig Count | N50 | Size | Protein Count | GC | GTDB classification |
| --- | --- | --- | --- | --- | --- | --- | --- | --- |
| GCA_013359425 | 0.9554 | 0.022 | 303 | 33,546 bp | 7,052,599 bp | 5711 | 0.5933 | d__Bacteria; p__KSB1; c__UBA2214; o__DRLW01; f__QEVD01; g__QEVD01; s__QEVD01 sp013359425 |
| GCA_013359385 | 0.884 | 0.011 | 1079 | 9,712 bp | 7,045,651 bp | 6732 | 0.5331 | Undefined (Failed Quality Check) |
| GCA_003576975 | 0.9224 | 0.033 | 778 | 14,736 bp | 7,248,468 bp | 6731 | 0.5287 | d__Bacteria; p__KSB1; c__UBA2214; o__DRLW01; f__QEVD01; g__QEVD01; s__QEVD01 sp003576975 |
| GCA_014338045 | 0.9114 | 0.0165 | 873 | 11,641 bp | 6,960,637 bp | 6435 | 0.5297 | d__Bacteria; p__KSB1; c__UBA2214; o__DRLW01; f__QEVD01; g__QEVD01; s__QEVD01 sp003576975 |
| GCA_008363195 | 0.9444 | 0.011 | 131 | 99,331 bp | 7,159,421 bp | 5936 | 0.5305 | d__Bacteria; p__KSB1; c__UBA2214; o__DRLW01; f__QEVD01; g__QEVD01; s__QEVD01 sp003576975 |
| GCA_013361015 | 0.7345 | 0.011 | 1166 | 6,621 bp | 6,481,920 bp | 6323 | 0.539 | Undefined (Failed Quality Check) |
| GCA_004357015 | 0.6687 | 0.0226 | 268 | 8,343 bp | 2,216,483 bp | 2127 | 0.5133 | d__Bacteria; p__KSB1; c__UBA2214; o__CR04bin15; f__CR04bin15; g__CR04bin15; s__CR04bin15 sp004357015 |
| GCA_011775825 | 0.8394 | 0.0598 | 1762 | 9,530 bp | 6,034,281 bp | 5955 | 0.4758 | Undefined (Failed Quality Check) |
| GCA_011777055 | 0.8394 | 0.0469 | 2009 | 9,170 bp | 6,337,027 bp | 6305 | 0.476 | Undefined (Failed Quality Check) |
| GCA_011774205 | 0.8394 | 0.0469 | 2009 | 9,170 bp | 6,337,027 bp | 6305 | 0.476 | Undefined (Failed Quality Check) |
| GCA_004356185 | 0.7631 | 0.044 | 332 | 7,784 bp | 2,588,190 bp | 2517 | 0.4372 | d__Bacteria; p__KSB1; c__UBA2214; o__CR04bin15; f__CR04bin15; g__N075bin58; s__N075bin58 sp004356825 |
| GCA_004356825 | 0.9444 | 0.033 | 358 | 13,543 bp | 4,364,253 bp | 3913 | 0.4453 | d__Bacteria; p__KSB1; c__UBA2214; o__CR04bin15; f__CR04bin15; g__N075bin58; s__N075bin58 sp004356825 |
| GCA_004356135 | 0.8235 | 0.044 | 329 | 10,731 bp | 3,324,124 bp | 3080 | 0.4426 | d__Bacteria; p__KSB1; c__UBA2214; o__CR04bin15; f__CR04bin15; g__N075bin58; s__N075bin58 sp004356825 |
| GCA_011040895 | 0.6217 | 0.022 | 469 | 6,504 bp | 2,663,704 bp | 2445 | 0.4414 | d__Bacteria; p__JdFR-76; c__JdFR-76; o__JdFR-76; f__DREG01; g__DREG01; s__DREG01 sp011040895 |
| GCA_013151735 | 0.9554 | 0.0006 | 262 | 25,296 bp | 3,950,189 bp | 3192 | 0.4796 | d__Bacteria; p__JdFR-76; c__JdFR-76; o__JdFR-76; f__BMS3Abin05; g__BMS3Abin05; s__BMS3Abin05 sp013151735 |
| GCA_002898095 | 0.9359 | 0.011 | 257 | 18,299 bp | 3,404,831 bp | 2871 | 0.4672 | d__Bacteria; p__JdFR-76; c__JdFR-76; o__JdFR-76; f__BMS3Abin05; g__BMS3Abin05; s__BMS3Abin05 sp002898015 |
| GCA_002898015 | 0.9884 | 0.011 | 148 | 37,008 bp | 3,627,982 bp | 2997 | 0.464 | d__Bacteria; p__JdFR-76; c__JdFR-76; o__JdFR-76; f__BMS3Abin05; g__BMS3Abin05; s__BMS3Abin05 sp002898015 |
| GCA_003644765 | 0.8543 | 0.033 | 496 | 6,025 bp | 2,648,335 bp | 2567 | 0.4324 | d__Bacteria; p__JdFR-76; c__JdFR-76; o__JdFR-76; f__4484-219; g__4484-219; s__4484-219 sp002085035 |
| GCA_003644775 | 0.9396 | 0.0504 | 434 | 7,895 bp | 2,780,181 bp | 2642 | 0.4282 | d__Bacteria; p__JdFR-76; c__JdFR-76; o__JdFR-76; f__4484-219; g__4484-219; s__4484-219 sp002085035 |
| GCA_003644785 | 0.7612 | 0.012 | 453 | 5,265 bp | 2,265,296 bp | 2172 | 0.4265 | d__Bacteria; p__JdFR-76; c__JdFR-76; o__JdFR-76; f__4484-219; g__4484-219; s__4484-219 sp002085035 |
| GCA_003645965 | 0.7777 | 0.0055 | 508 | 5,049 bp | 2,420,289 bp | 2406 | 0.4302 | d__Bacteria; p__JdFR-76; c__JdFR-76; o__JdFR-76; f__4484-219; g__4484-219; s__4484-219 sp002085035 |
| GCA_002085035 | 0.646 | 0.011 | 156 | 9,752 bp | 1,433,169 bp | 1415 | 0.4248 | d__Bacteria; p__JdFR-76; c__JdFR-76; o__JdFR-76; f__4484-219; g__4484-219; s__4484-219 sp002085035 |
| GCA_014729735 | 0.8473 | 0.0486 | 823 | 6,544 bp | 4,842,997 bp | 4400 | 0.4284 | d__Bacteria; p__KSB1; c__UBA2214; o__AABM5-25-91; f__JAFGDW01; g__WJJN01; s__WJJN01 sp014729735 |
| GCA_002085355 | 0.7253 | 0.0116 | 275 | 10,413 bp | 2,720,207 bp | 2429 | 0.4287 | d__Bacteria; p__KSB1; c__UBA2214; o__AABM5-25-91; f__AABM5-25-91; g__4484-87; s__4484-87 sp002085355 |
| GCA_002084535 | 0.5813 | 0.011 | 258 | 8,998 bp | 2,260,459 bp | 1975 | 0.3848 | d__Bacteria; p__KSB1; c__UBA2214; o__AABM5-25-91; f__AABM5-25-91; g__NBLI01; s__NBLI01 sp002084535 |
| GCA_014728115 | 0.9554 | 0.044 | 338 | 40,493 bp | 6,960,450 bp | 5656 | 0.46 | d__Bacteria; p__KSB1; c__UBA2214; o__UBA2214; f__WJMF01; g__WJME01; s__WJME01 sp014728115 |
| GCA_014728085 | 0.9554 | 0.022 | 244 | 43,030 bp | 5,189,837 bp | 4157 | 0.4597 | d__Bacteria; p__KSB1; c__UBA2214; o__UBA2214; f__WJMF01; g__WJMF01; s__WJMF01 sp014728085 |
| GCA_001771235 | 0.851 | 0 | 1329 | 6,572 bp | 3,937,874 bp | 3575 | 0.489 | Undefined (Failed Quality Check) |
| GCA_003854995 | 0.7576 | 0.033 | 661 | 8,787 bp | 3,541,199 bp | 3285 | 0.5265 | d__Bacteria; p__KSB1; c__UBA2214; o__UBA2214; f__Zgenome-0027; g__Zgenome-0027; s__Zgenome-0027 sp003854995 |
| GCA_003854975 | 0.9005 | 0.0659 | 305 | 78,315 bp | 5,417,862 bp | 4326 | 0.4683 | d__Bacteria; p__KSB1; c__UBA2214; o__UBA2214; f__Zgenome-0027; g__Zgenome-0027; s__Zgenome-0027 sp003854975 |
| GCA_003818595 | 0.8622 | 0.0464 | 979 | 5,811 bp | 4,816,135 bp | 4579 | 0.4802 | d__Bacteria; p__KSB1; c__UBA2214; o__UBA2214; f__Zgenome-0027; g__RPQV01; s__RPQV01 sp003818595 |
| GCA_016214895 | 1 | 0 | 26 | 429,255 bp | 3,211,286 bp | 2593 | 0.6005 | d__Bacteria; p__AABM5-125-24; c__RPQS01; o__RPQS01; f__RPQS01; g__JACRMR01; s__JACRMR01 sp016214895 |
| GCA_003818605 | 0.9554 | 0.0824 | 253 | 21,372 bp | 3,228,723 bp | 2829 | 0.5352 | d__Bacteria; p__AABM5-125-24; c__RPQS01; o__RPQS01; f__RPQS01; g__RPQS01; s__RPQS01 sp003818605 |
| GCA_018825465 | 0.9176 | 0.0126 | 439 | 9,405 bp | 3,220,104 bp | 2946 | 0.5812 | d__Bacteria; p__AABM5-125-24; c__RPQS01; o__RPQS01; f__RPQS01; g__JAHJDG01; s__JAHJDG01 sp018812265 |
| GCA_018812265 | 0.978 | 0 | 235 | 23,311 bp | 3,496,761 bp | 3005 | 0.5786 | d__Bacteria; p__AABM5-125-24; c__RPQS01; o__RPQS01; f__RPQS01; g__JAHJDG01; s__JAHJDG01 sp018812265 |
| GCA_016703065 | 0.9609 | 0 | 9 | 545,695 bp | 3,088,792 bp | 2950 | 0.5627 | d__Bacteria; p__AABM5-125-24; c__RPQS01; o__RPQS01; f__RPQS01; g__JABWCQ01; s__JABWCQ01 sp016703065 |
| GCA_013360195 | 0.9884 | 0 | 46 | 167,035 bp | 3,232,388 bp | 2727 | 0.5243 | d__Bacteria; p__AABM5-125-24; c__RPQS01; o__RPQS01; f__RPQS01; g__JABWCQ01; s__JABWCQ01 sp013360195 |
| GCA_016721565 | 0.9499 | 0 | 62 | 84,879 bp | 3,716,885 bp | 3855 | 0.5133 | d__Bacteria; p__AABM5-125-24; c__RPQS01; o__RPQS01; f__RPQS01; g__JABWCQ01; s__JABWCQ01 sp016721565 |
| GCA_016702305 | 0.989 | 0 | 17 | 807,450 bp | 3,383,257 bp | 2946 | 0.5772 | d__Bacteria; p__AABM5-125-24; c__RPQS01; o__RPQS01; f__RPQS01; g__JABWCQ01; s__JABWCQ01 sp016702305 |
| GCA_016712825 | 0.967 | 0.011 | 52 | 392,969 bp | 4,294,646 bp | 4301 | 0.5703 | d__Bacteria; p__AABM5-125-24; c__RPQS01; o__RPQS01; f__RPQS01; g__JABWCQ01; s__JABWCQ01 sp016702305 |
| GCA_018822485 | 0.8785 | 0.0378 | 469 | 9,362 bp | 3,254,808 bp | 2943 | 0.5184 | d__Bacteria; p__AABM5-125-24; c__RPQS01; o__RPQS01; f__RPQS01; g__JAHIZR01; s__JAHIZR01 sp018814465 |
| GCA_018824745 | 0.7303 | 0 | 528 | 5,835 bp | 2,647,184 bp | 2500 | 0.5206 | d__Bacteria; p__AABM5-125-24; c__RPQS01; o__RPQS01; f__RPQS01; g__JAHIZR01; s__JAHIZR01 sp018814465 |
| GCA_018814465 | 0.894 | 0.0065 | 513 | 8,236 bp | 3,288,253 bp | 3008 | 0.5184 | d__Bacteria; p__AABM5-125-24; c__RPQS01; o__RPQS01; f__RPQS01; g__JAHIZR01; s__JAHIZR01 sp018814465 |
| GCA_003599535 | 0.9121 | 0.011 | 140 | 31,990 bp | 3,770,623 bp | 3113 | 0.6297 | d__Bacteria; p__AABM5-125-24; c__B3-LCP; o__B3-LCP; f__B3-LCP; g__SURF-9; s__SURF-9 sp003599535 |
| GCA_018812585 | 0.9615 | 0.033 | 383 | 16,035 bp | 3,847,866 bp | 3401 | 0.4928 | d__Bacteria; p__AABM5-125-24; c__B3-LCP; o__B3-LCP; f__B3-LCP; g__JAHJDJ01; s__JAHJDJ01 sp018812585 |
| GCA_005223185 | 0.978 | 0.001 | 18 | 359,442 bp | 3,794,239 bp | 2993 | 0.4654 | d__Bacteria; p__AABM5-125-24; c__B3-LCP; o__B3-LCP; f__B3-LCP; g__B3-LCP; s__B3-LCP sp005223185 |
| GCA_016783645 | 0.7363 | 0.033 | 221 | 13,719 bp | 2,725,362 bp | 2303 | 0.4515 | d__Bacteria; p__AABM5-125-24; c__JABMRZ01; o__JABMRZ01; f__JADHWD01; g__JADHWD01; s__JADHWD01 sp016783645 |
| GCA_013202315 | 0.7991 | 0.0201 | 557 | 7,131 bp | 3,345,940 bp | 2709 | 0.4596 | d__Bacteria; p__AABM5-125-24; c__JABMRZ01; o__JABMRZ01; f__JABMRZ01; g__JABMRZ01; s__JABMRZ01 sp013202315 |
| GCA_016867835 | 0.978 | 0 | 214 | 21,061 bp | 2,903,125 bp | 2518 | 0.5793 | d__Bacteria; p__AABM5-125-24; c__AABM5-125-24; o__AABM5-125-24; f__AABM5-125-24; g__VGIQ01; s__VGIQ01 sp016867835 |
| GCA_016867855 | 0.9719 | 0.022 | 294 | 14,696 bp | 3,099,451 bp | 2701 | 0.5088 | d__Bacteria; p__AABM5-125-24; c__AABM5-125-24; o__AABM5-125-24; f__AABM5-125-24; g__VGIO01; s__VGIO01 sp016867855 |
| GCA_013202285 | 0.9426 | 0.011 | 261 | 16,143 bp | 3,005,082 bp | 2372 | 0.4839 | d__Bacteria; p__AABM5-125-24; c__AABM5-125-24; o__AABM5-125-24; f__AABM5-125-24; g__JABMRV01; s__JABMRV01 sp013202285 |
| GCA_018644935 | 0.9609 | 0.0165 | 217 | 35,462 bp | 4,536,430 bp | 3220 | 0.429 | d__Bacteria; p__AABM5-125-24; c__AABM5-125-24; o__AABM5-125-24; f__AABM5-125-24; g__JABGPL01; s__JABGPL01 sp018644935 |
| GCA_018675255 | 0.8381 | 0.044 | 804 | 7,213 bp | 4,679,327 bp | 3977 | 0.424 | d__Bacteria; p__AABM5-125-24; c__AABM5-125-24; o__AABM5-125-24; f__AABM5-125-24; g__JABGPL01; s__JABGPL01 sp018644935 |
| GCA_018676415 | 0.9389 | 0.0171 | 472 | 15,655 bp | 4,905,963 bp | 3791 | 0.4252 | d__Bacteria; p__AABM5-125-24; c__AABM5-125-24; o__AABM5-125-24; f__AABM5-125-24; g__JABGPL01; s__JABGPL01 sp018644935 |
| GCA_018263585 | 0.967 | 0 | 165 | 24,817 bp | 2,845,983 bp | 2203 | 0.6609 | d__Bacteria; p__AABM5-125-24; c__BMS3BBIN04; o__BMS3BBIN04; f__BMS3BBIN04; g__JAANWZ01; s__JAANWZ01 sp018263585 |
| GCA_002898195 | 0.7037 | 0.0055 | 512 | 5,228 bp | 2,496,221 bp | 2351 | 0.5095 | d__Bacteria; p__AABM5-125-24; c__BMS3BBIN04; o__BMS3BBIN04; f__BMS3BBIN04; g__BMS3BBIN04; s__BMS3BBIN04 sp002898195 |
| GCA_014729955 | 0.8851 | 0.0251 | 612 | 6,754 bp | 3,642,132 bp | 3159 | 0.5736 | d__Bacteria; p__AABM5-125-24; c__BMS3BBIN04; o__BMS3BBIN04; f__BMS3BBIN04; g__WJJA01; s__WJJA01 sp014729955 |
| GCF_000018785 | 0.9865 | 0 | 1 | 1,496,992 bp | 1,496,992 bp | 1389 | 0.3193 | d__Bacteria; p__Firmicutes; c__Bacilli; o__Acholeplasmatales; f__Acholeplasmataceae; g__Acholeplasma; s__Acholeplasma laidlawii |
| GCF_000007925 | 1 | 0 | 1 | 1,751,080 bp | 1,751,080 bp | 1908 | 0.3644 | d__Bacteria; p__Cyanobacteria; c__Cyanobacteriia; o__PCC-6307; f__Cyanobiaceae; g__Prochlorococcus; s__Prochlorococcus marinus |
| GCF_000011385 | 0.9915 | 0.0085 | 1 | 4,659,019 bp | 4,659,019 bp | 4517 | 0.62 | d__Bacteria; p__Cyanobacteria; c__Cyanobacteriia; o__Gloeobacterales; f__Gloeobacteraceae; g__Gloeobacter; s__Gloeobacter violaceus |
| GCF_000020025 | 0.9956 | 0.0044 | 6 | 8,234,322 bp | 9,059,191 bp | 7754 | 0.4135 | d__Bacteria; p__Cyanobacteria; c__Cyanobacteriia; o__Cyanobacteriales; f__Nostocaceae; g__Nostoc; s__Nostoc punctiforme |
| GCF_000010625 | 0.9989 | 0.0051 | 1 | 5,842,795 bp | 5,842,795 bp | 5605 | 0.4233 | d__Bacteria; p__Cyanobacteria; c__Cyanobacteriia; o__Cyanobacteriales; f__Microcystaceae; g__Microcystis; s__Microcystis aeruginosa |
| GCF_000010065 | 0.9973 | 0 | 1 | 2,696,255 bp | 2,696,255 bp | 2691 | 0.5548 | d__Bacteria; p__Cyanobacteria; c__Cyanobacteriia; o__Synechococcales; f__Synechococcaceae; g__Synechococcus; s__Synechococcuselongatus |

**
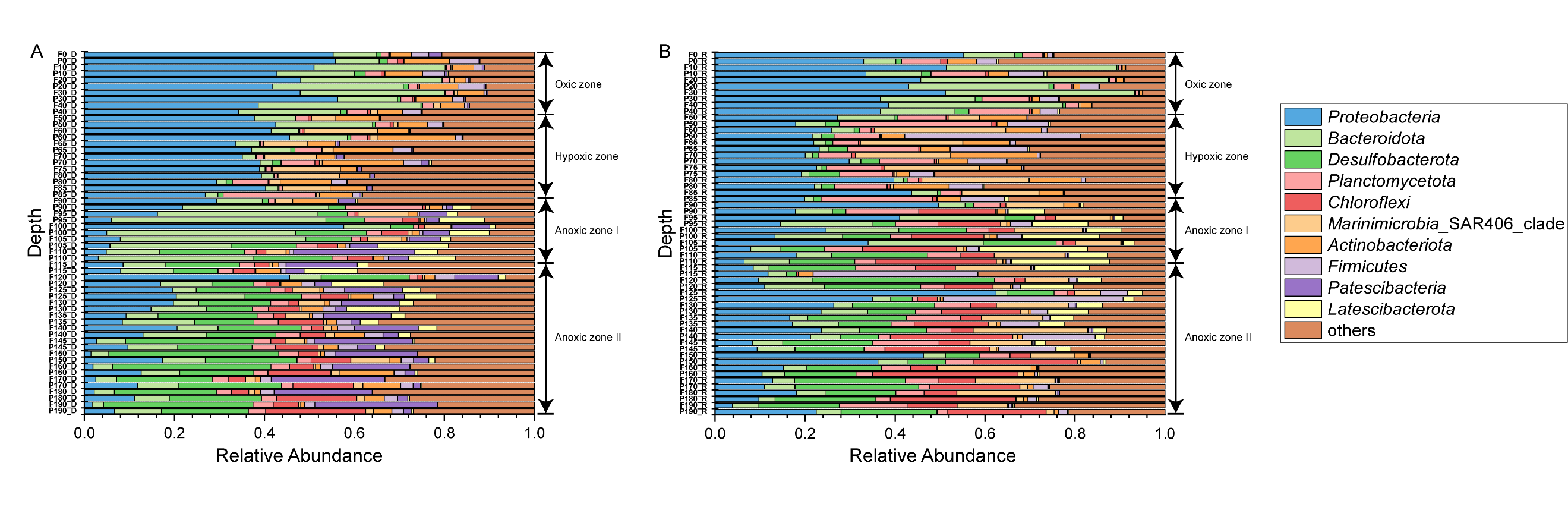
**

**Figure S1. Profiles of microbial communities at phylum level in DNA samples (A) and RNA samples (B).**

**
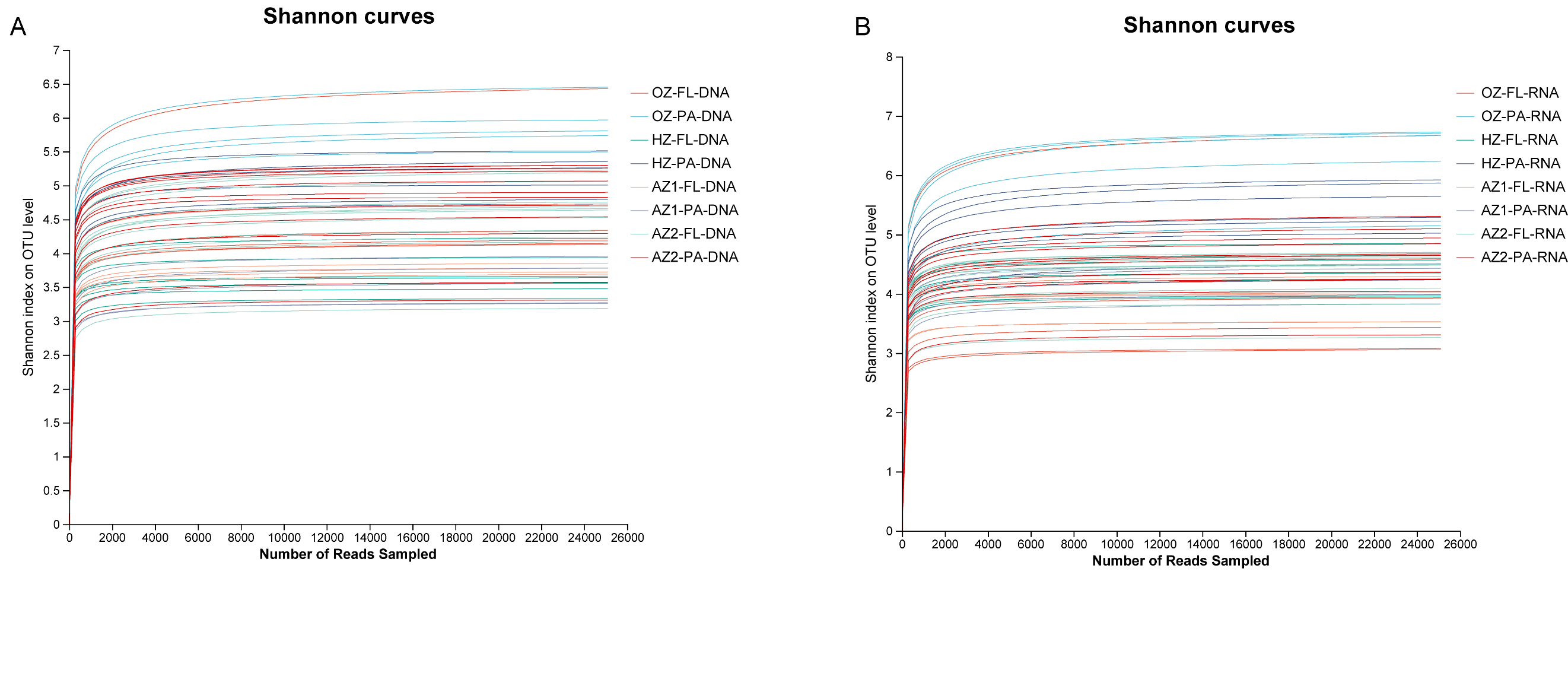
**

**Figure S2. The rarefaction curve of shannon index in DNA samples (A) and RNA samples (B).**

**
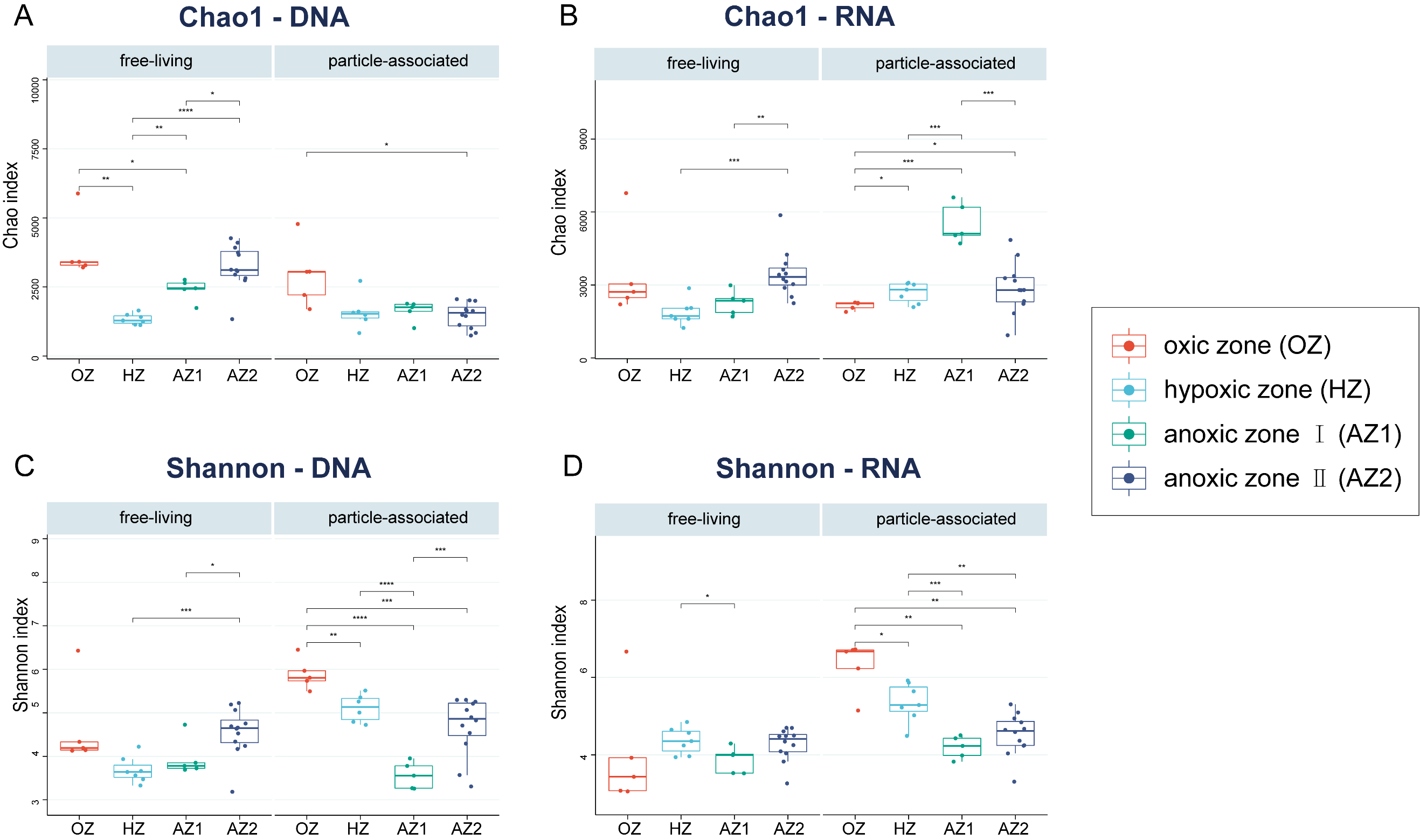
**

**Figure S3. Chao1 and Shannon indices among the total communities by depth and lifestyle in the SYBH.** Chao1 on (A) DNA and (B) RNA levels; Shannon on (C) DNA and (D) RNA levels. The between-group variation adopts the t.test (*, P < 0.1; **, P < 0.01; ***, P < 0.001).

**
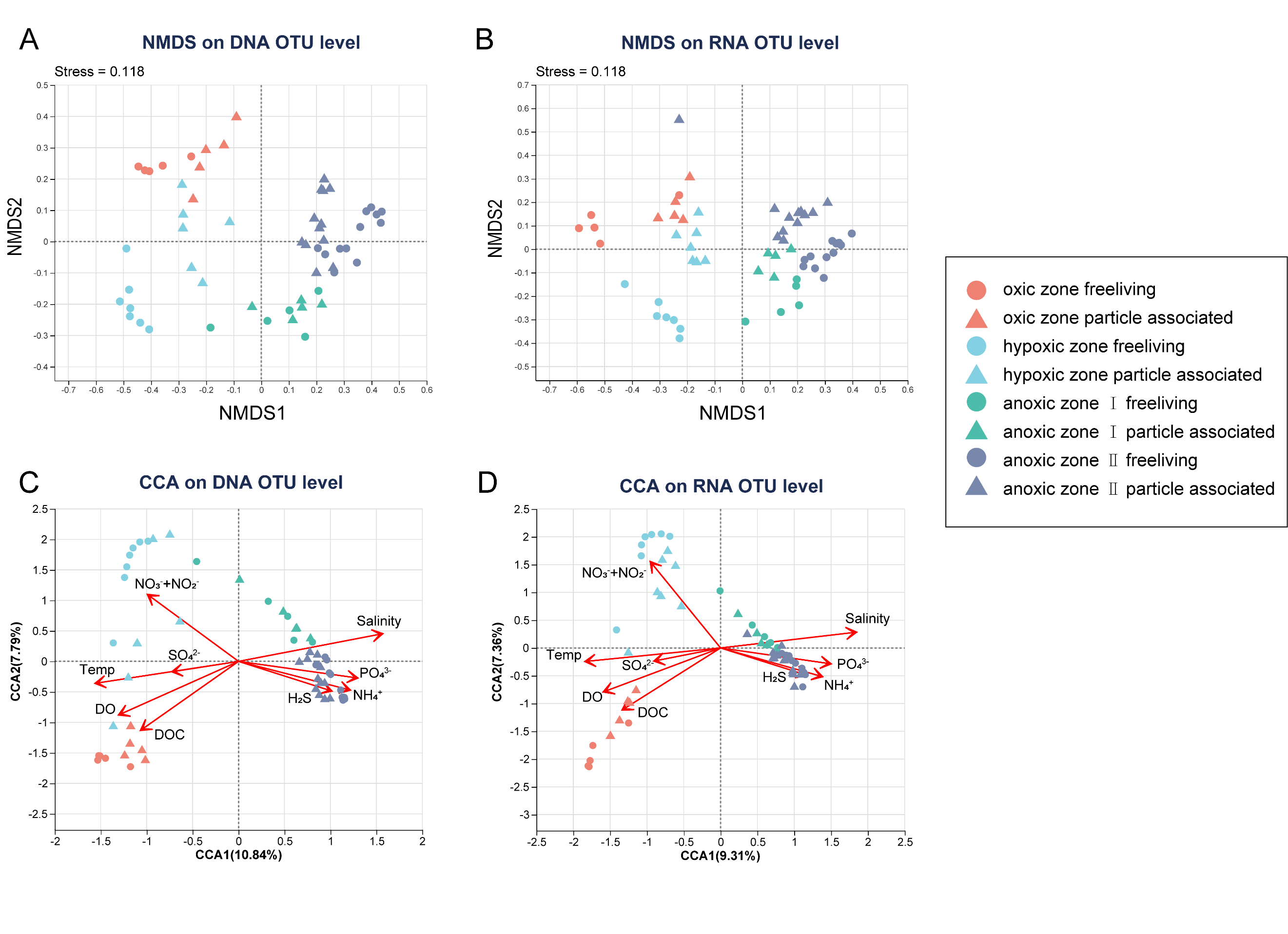
Figure S4. Community dissimilarity analysis among the total communities.** (A, B) NMDS analysis illustrating the differences between groups. The distance algorithm adopts Bray-Curtis dissimilarity, and the between-group variation adopts Adonis. (C, D) CCA illustrating the relationships between microbial communities and environmental factors. DO [r^2^ (DNA) = 0.8856, r^2^ (RNA) = 0.8567], DOC [r^2^ (DNA) = 0.8446, r^2^ (RNA) = 0.81], Salinity [r^2^ (DNA) = 0.9734, r^2^ (RNA) = 0.9592], Temp [r^2^ (DNA) = 0.9374, r^2^ (RNA) = 0.9494], NO_3_^−^ and NO_2_^−^ [r^2^ (DNA) = 0.747, r^2^ (RNA) = 0.8789], SO_4_^2-^ [r^2^ (DNA) = 0.1918, r^2^ (RNA) = 0.228], H_2_S [r^2^ (DNA) = 0.4421, r^2^ (RNA) = 0.4301], NH_4_^+^ [r^2^ (DNA) = 0.6035, r^2^ (RNA) = 0.5917] and PO_4_^3-^ [r^2^ (DNA) = 0.6309, r^2^ (RNA) = 0.6355]. Temp, temperature; DO, dissolved oxygen; DOC, dissolved organic carbon.


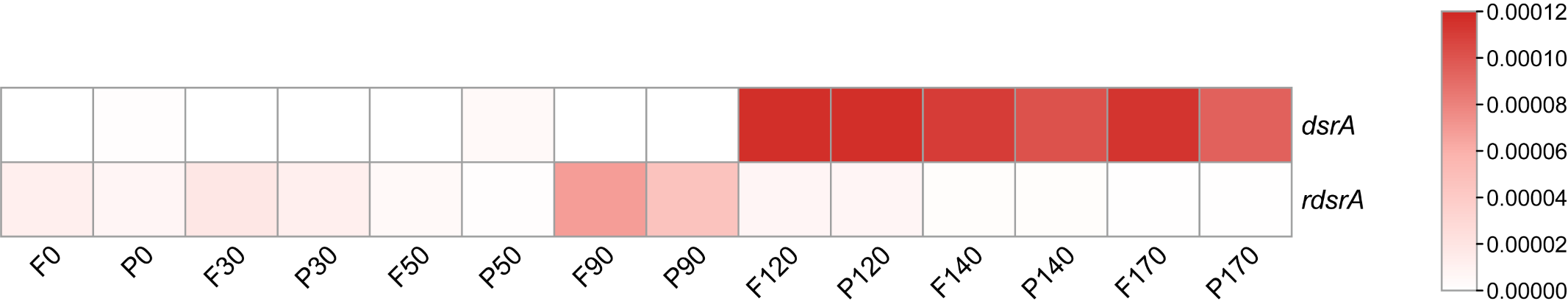


**Figure S5. Relative abundances of *dsrA* and *rdsrA* at different depths.**

**
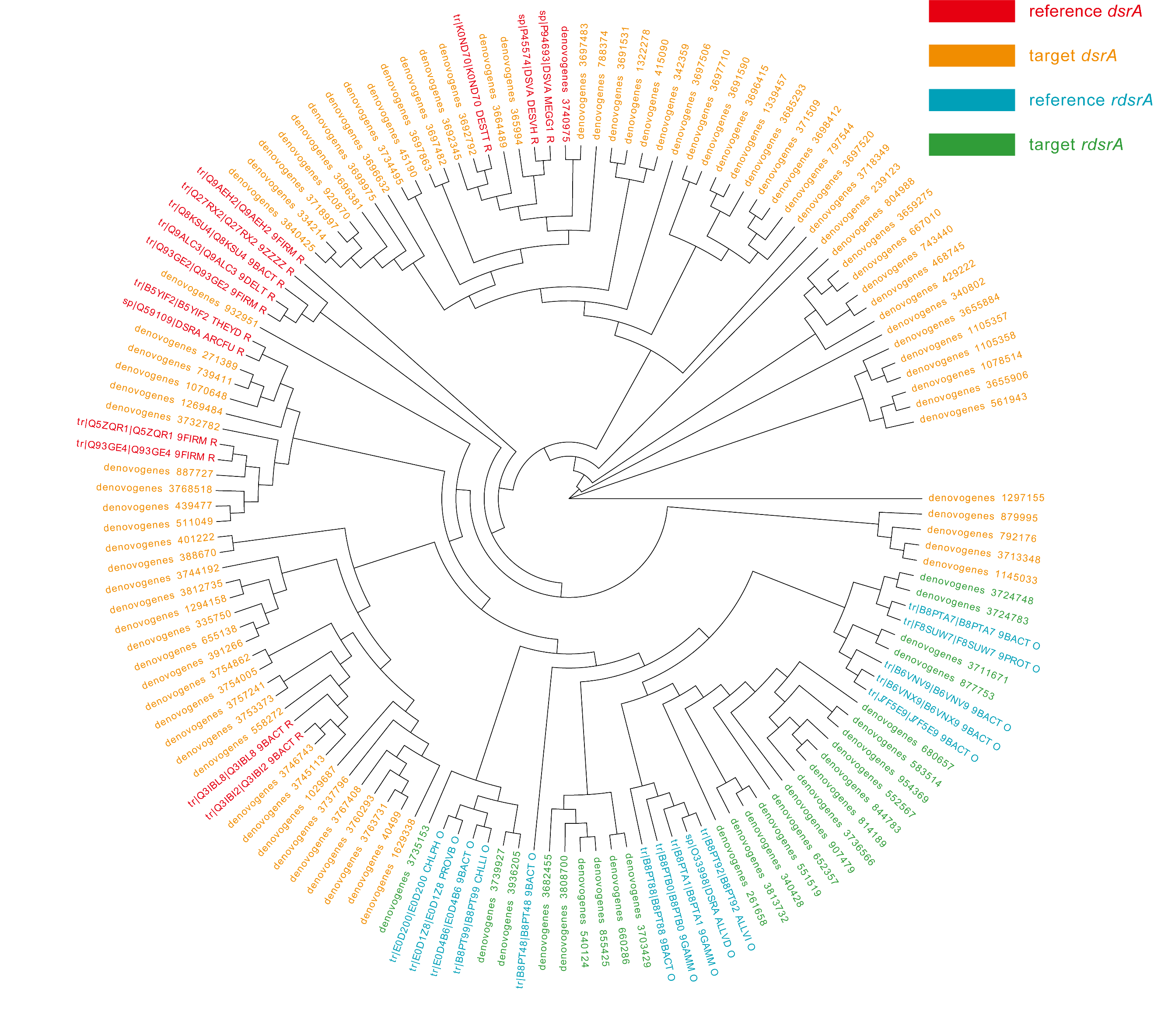
**

**Figure S6. Phylogenetic tree of *dsrA* and *rdsrA* retrieved from the SYBH metagenomes.** Reference sequences were downloaded from NCBI.


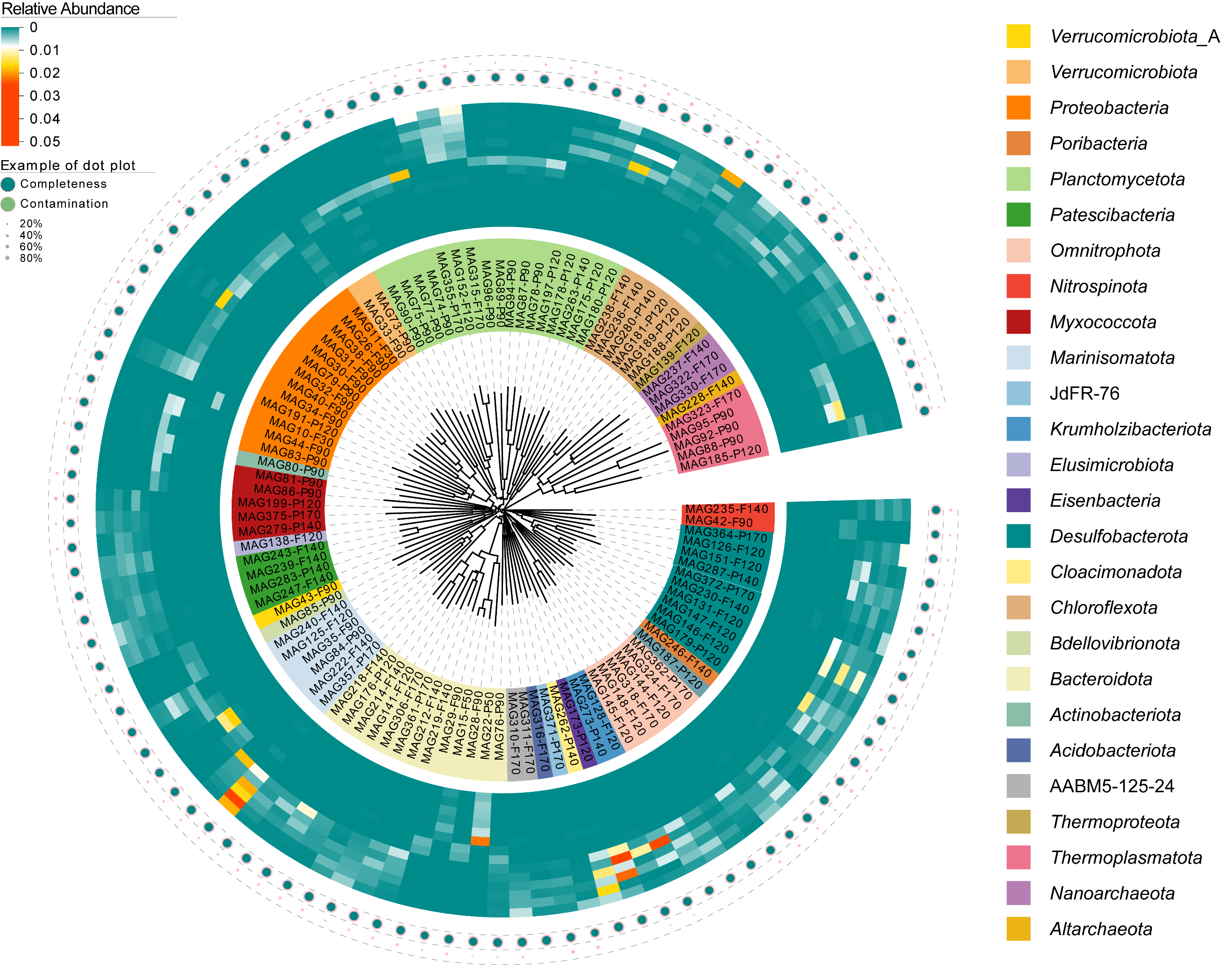


**Figure S7. Phylogenetic tree of 108 MAGs in the SYBH based on the single-copy core sequences.**


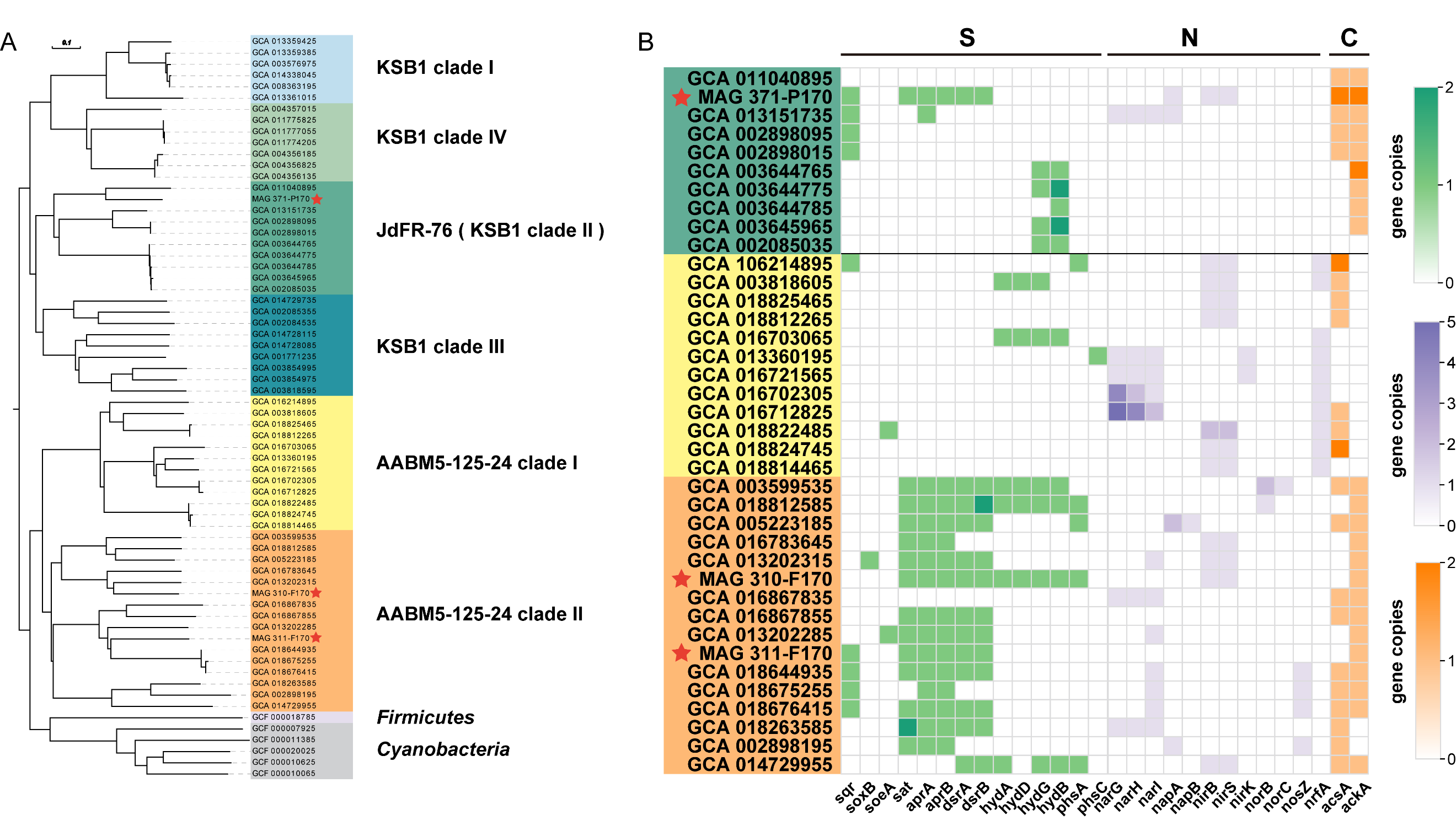


**Figure S8. Phylogenomic analysis and metabolic gene distribution of JdFR-76 and AABM-125-24 MAGs.** (A) Maximum likelihood phylogenetic tree of MAG371, MAG310, MAG311 and reference genomes. The MAGs from this study were marked with a red star; (B) Distribution of genes involved in carbon, nitrogen, sulfur metabolisms predicted in these genomes.

**Reference**

1. Yu X, Zhou J, Song W, Xu M, He Q, Peng Y, Tian Y, Wang C, Shu L, Wang S, Yan Q, Liu J, Tu Q, He Z. 2021. SCycDB: A curated functional gene database for metagenomic profiling of sulphur cycling pathways. *Molecular Ecology Resources* 21:924–940.

2. Li Q, Zhou Y, Lu R, Zheng P, Wang Y. 2022. Phylogeny, distribution and potential metabolism of candidate bacterial phylum KSB1. *PeerJ* 10:e13241.

3. Müller AL, Kjeldsen KU, Rattei T, Pester M, Loy A. 2015. Phylogenetic and environmental diversity of DsrAB-type dissimilatory (bi)sulfite reductases. *ISME J* 9:1152–1165.
